# Structural basis of transcription-coupled H3K36 trimethylation by Set2 in coordination with FACT

**DOI:** 10.1126/sciadv.aed1952

**Published:** 2026-01-28

**Authors:** Tomoya Kujirai, Haruhiko Ehara, Tomoko Ito, Masami Henmi, Eriko Oya, Takehiko Kobayashi, Shun-ichi Sekine, Hitoshi Kurumizaka

**Affiliations:** ^1^ Laboratory of Chromatin Structure and Function, Institute for Quantitative Biosciences, The University of Tokyo, 1-1-1 Yayoi, Bunkyo-ku, Tokyo 113-0032, Japan.; ^2^ Laboratory for Transcription Structural Biology, RIKEN Center for Integrative Medical Sciences, 1-7-22 Suehiro-cho, Tsurumi-ku, Yokohama 230-0045, Japan.; ^3^ Laboratory of Genome Regeneration, Institute for Quantitative Biosciences, The University of Tokyo, 1-1-1 Yayoi, Bunkyo-ku, Tokyo 113-0032, Japan.; ^4^ Department of Biological Sciences, Graduate School of Science, The University of Tokyo, 1-1-1 Yayoi, Bunkyo-ku, Tokyo 113-0032, Japan.

## Abstract

Trimethylation of the histone H3K36 residue (H3K36me3) plays an indispensable role in ensuring transcription fidelity by suppressing undesired cryptic transcription in chromatin. H3K36me3 modification is accomplished by Set2/SETD2 during transcription elongation by the RNA polymerase II elongation complex (EC). Here, we found that Set2-mediated H3K36me3 deposition occurs on the nucleosome reassembling behind the EC. The histone chaperone FACT suppresses H3K36me3 deposition on the downstream nucleosome, thereby ensuring that Set2 targets specifically on the reassembling upstream nucleosome. Cryo–electron microscopy structures of the nucleosome-transcribing EC complexed with Set2 revealed that Set2 is anchored by the Spt6 subunit of the EC to capture both of the H3 N-terminal tails in a stepwise manner during the nucleosome reassembly process. Abrogation of the Set2-EC interaction leads to defective transcription-coupled H3K36me3 deposition. These insights elucidate the structure-based mechanism of transcription-coupled H3K36me3 deposition in chromatin.

## Teaser

Cryo-EM structures of RNAPII EC-Set2-nucleosome with FACT reveal the mechanism of transcription-coupled H3K36 trimethylation.

## INTRODUCTION

The nucleosome is composed of two each of four histones, H2A, H2B, H3, and H4, and 145 to 147 base pairs (bp) of DNA as the basic unit of chromatin in eukaryotes and plays an essential role in the compaction of genomic DNA (*1*). The nucleosome also functions as a regulator of genome activities, such as transcription, replication, recombination, and repair (*2*–*4*). In transcription, the nucleosome suppresses the inappropriate production of RNA molecules by RNA polymerases and also modulates the gene expression level by suppressing transcription processes (*3*, *5*). In chromatin, transcription fidelity is ensured by the trimethylation of the Lys^36^ residue in the N-terminal tail of histone H3 (H3K36me3), which plays an important role in suppressing undesired cryptic transcription (*6*–*9*). In addition, H3K36me3 reportedly regulates DNA methylation, histone acetylation, chromatin remodeling, RNA processing, and DNA repair in the genome (*10*–*12*). Yeast Set2 (SETD2 in higher eukaryotes) promotes H3K36me3 deposition during the transcription elongation process by RNA polymerase II (RNAPII) (*13*–*16*).

To produce nascent RNAs in chromatin, RNAPII transcribes the DNA wrapped around the histone core in nucleosomes by incrementally uncoiling the nucleosomal DNA until its center was reached {superhelical location (0) [SHL(0)]} (*17*–*19*). The nucleosome is then disassembled when RNAPII passes through the SHL(0) position of the nucleosome (*20*). Transcription elongation factors, Spt4/5, Elf1, Spt6, Spn1, and Paf1C, associate with RNAPII to form the RNAPII elongation complex (EC). The EC exhibits substantially enhanced activity for nucleosome transcription (*18*, *20*–*23*). After the disassembly process, the nucleosome is readily reassembled by histone transfer to the upstream DNA behind the transcribing EC with the aid of the histone chaperone FACT (*20*). This EC/FACT-mediated nucleosome reassembly occurs on the EC upstream surface, the “cradle” formed by Spt4, Spt5, Spt6, and the Paf1C subunits, Leo1 and Rtf1, on the rim of the DNA exit of RNAPII (*20*).

The Set2 methyltransferase catalyzes H3K36me3 deposition in the nucleosome during the transcription elongation process by the EC (*13*–*16*). Set2 consists of several conserved domains, including the catalytic (AWS, SET, and Post-SET) domain, AID (autoinhibitory domain), WW domain, CC (coiled-coil) domain, and SRI (Set2 Rpb1-interacting) domain (*12*, *24*). The catalytic activity is reportedly regulated by the AID, which is modulated by the SRI domain binding to the phosphorylated C-terminal domain of RNAPII (*25*–*27*). A previous genetic study suggested that H3K36me3 deposition by Set2 is dependent on Spt6 (*28*). However, the mechanism by which Set2 specifically deposits H3K36me3 in the nucleosome in a transcription-coupled manner has remained enigmatic.

In the present study, we performed a detailed biochemical analysis of transcription-coupled H3K36me3 deposition by Set2 and found that Set2 introduces histone modification primarily during the nucleosome reassembly process on the upstream side of the EC, in coordination with FACT. Accompanying cryo–electron microscopy (cryo-EM) analyses of the nucleosome-transcribing EC complexed with Set2 demonstrated that Set2 is anchored to Spt6, so it can capture a rewrapping nucleosome intermediate on the upstream side of the EC to catalyze the methylation. These structures reveal two distinct modes of H3K36me3 deposition (“on-cradle” and “off-cradle”), accounting for the mechanism by which Set2 promotes H3K36me3 deposition on both copies of H3 within the nucleosome one by one, during nucleosome traversal by the EC.

## RESULTS

### Transcription elongation and FACT are required for proper nucleosomal H3K36me3 deposition by Set2

**Fig. 1.**
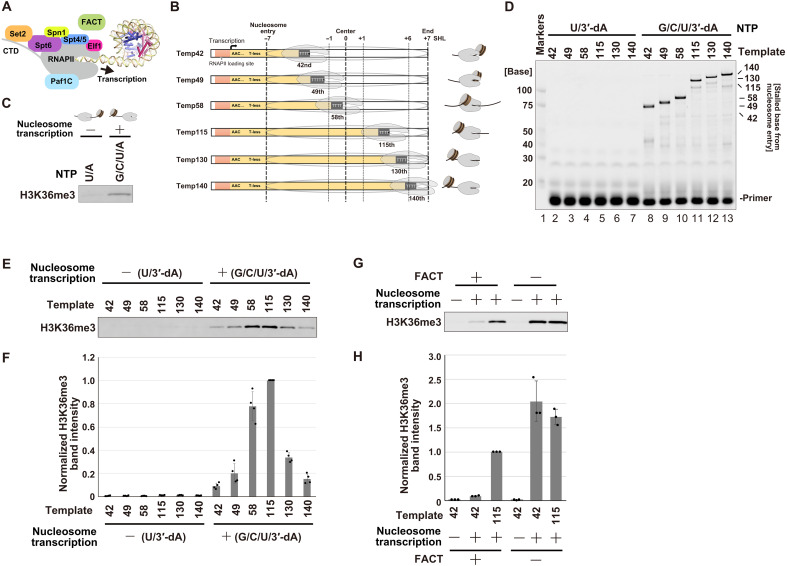
Transcription-coupled H3K36me3 deposition by Set2. (**A**) Schematic representation of in vitro transcription on the nucleosome template. (**B**) Nucleosome templates used in the H3K36me3 deposition assay. Cartoons depicting the relative position of a nucleosome to RNAPII are shown. (**C**) H3K36me3 deposition with (G/C/U/A) or without (U/A) transcription through a nucleosome in Temp42. This experiment was performed in triplicate. (**D**) Urea-PAGE analysis of the elongated RNA by EC with [G/C/U/3′-dATP (3′-dA)] or without (U/3′-dATP) nucleosome transcription. (**E**) Western blot of H3K36me3 deposition in different nucleosome templates. (**F**) Quantification of (E). (**G**) Western blot of H3K36me3 deposition with (G/C/U/3′-dATP) or without (U/3′-dATP) nucleosome transcription in Temp42 and Temp115 in the presence or absence of FACT. (**H**) Quantification of (G). The means (bars) and SD (error bars) of the relative values of the H3K36me3 signal intensities compared to that of Temp115 with G/C/U/3′-dATP of three (H) or four (F) independent experiments are shown.

To study transcription-coupled H3K36me3 deposition by Set2, we first established an in vitro cotranscriptional methylation assay on the basis of the nucleosomal transcription system using the yeast *Komagataella phaffii* (formerly *K. pastoris*) EC (*20*). The transcription reaction was conducted in the presence of the elongation factors TFIIS, Spt4/5, Elf1, Spt6, Spn1, and Paf1C (Paf1, Leo1, Ctr9, Cdc73, and Rtf1) and the histone chaperone FACT, which are important for transcription elongation over the nucleosome (Fig. 1A and fig. S1A). We prepared six nucleosome templates, termed Temp42, Temp49, Temp58, Temp115, Temp130, and Temp140, each containing a poly-T track at positions 42, 49, 58, 115, 130, and 140 bp from the nucleosome entry site, respectively (Fig. 1B and fig. S1, B and C). We then conducted nucleosome transcription in the presence of a stoichiometric amount of Set2 (Set2:RNAPII = 1:1) and *S*-adenosylmethionine (SAM), a methyl donor.

In these reactions, when transcription elongation was blocked after two bases of EC progression, by omitting guanosine 5′-triphosphate (GTP) and cytidine 5′-triphosphate (CTP), only a small amount of H3K36me3 was detected regardless of the template DNA used (Fig. 1C, lane U/A). With all four nucleoside triphosphates, H3K36me3 deposition was efficiently promoted by Set2 (Fig. 1C, lane G/C/U/A). These results indicate that H3K36 trimethylation can only occur cotranscriptionally, and the close interplay between the EC and the nucleosome is critical for the Set2 activity.

To clarify the timing of H3K36me3 deposition during nucleosome transcription by the EC, we conducted the transcription-coupled H3K36me3 deposition assay with the Temp42, Temp49, Temp58, Temp115, Temp130, and Temp140 nucleosomes. The transcription reaction was performed in the presence of 3′-deoxyadenosine triphosphate (3′-dATP) instead of ATP (adenosine 5′-triphosphate) so that transcription elongation was stalled at the poly-T track. Consequently, transcription elongation was efficiently promoted, and the EC stalled when the RNAPII catalytic center reached the 42-, 49-, 58-, 115-, 130-, and 140-bp positions, where the leading edge of RNAPII is located at the SHL(−1), SHL(0), SHL(+1), SHL(+6), SHL(+7.5), and SHL(+8.5) positions on the nucleosomal DNA, respectively (Fig. 1D, lanes 8 to 13). Notably, compared to the Temp42 or Temp49 substrate, H3K36me3 deposition was substantially enhanced with the Temp58 substrate (Fig. 1, E and F) and even more prominent with the Temp115 substrate. In our previous study, at the 42- and 49-bp positions, the disassembling nucleosome was observed on the downstream side of the EC (*20*). In contrast, at the 58- and 115-bp positions, the (sub)nucleosome was already transferred to the upstream side of the EC. H3K36me3 deposition in the Temp115 nucleosome was not promoted when either Set2 or EC was omitted from the reaction mixture (fig. S2, A to C). Therefore, Set2-mediated H3K36me3 deposition is EC-dependent and likely promoted more efficiently on the reassembling nucleosome on the upstream side of the EC rather than on the one remaining on the downstream side of the EC. H3K36me3 deposition on the Temp130 and Temp140 substrates was decreased as compared to that on the Temp115 substrate. This suggests that the Temp115 substrate may provide a more favorable intermediate reassembly state of the nucleosome for transcription-coupled H3K36me3 deposition by Set2 (discussed later).

The transcription-coupled H3K36me3 deposition reaction was conducted in the presence of FACT, phosphorylated by casein kinase 2 (CK2). Unexpectedly, under the conditions without FACT, the downstream nucleosome (Temp42) was efficiently methylated, thereby losing the upstream specificity of H3K36me3 deposition (Fig. 1, G and H). These results suggest that FACT suppresses H3K36me3 deposition by Set2 on the downstream nucleosome, ensuring the tight coupling of histone modification with transcription elongation.

It should be noted that Set2/SETD2 (mammalian homolog) reportedly mediates H3K36me3 deposition in the nucleosome without RNAPII and elongation factors in vitro (*6*, *29*, *30*). Consistently, our nucleosomal H3K36me3 deposition assay also detected the Set2 methyltransferase activity in the presence of Set2 and nucleosome without EC and FACT (fig. S2, D to H). However, H3K36me3 deposition was barely detectable, when H3K36 methylation was examined in the presence of RNAPII and elongation factors, but limited nucleoside triphosphates (U/3′-dATP; Fig. 1, D and E). This discrepancy may be explained by the possibility that Set2 is sequestered from the downstream nucleosome by the EC, which is distant from the nucleosome. Efficient H3K36me3 deposition may require transcription elongation that accompanies nucleosome reassembly in proximity to Set2 tethered by the EC.

### Structures of EC115-Set2 complexes (“on-cradle” structures)

**Fig. 2.**
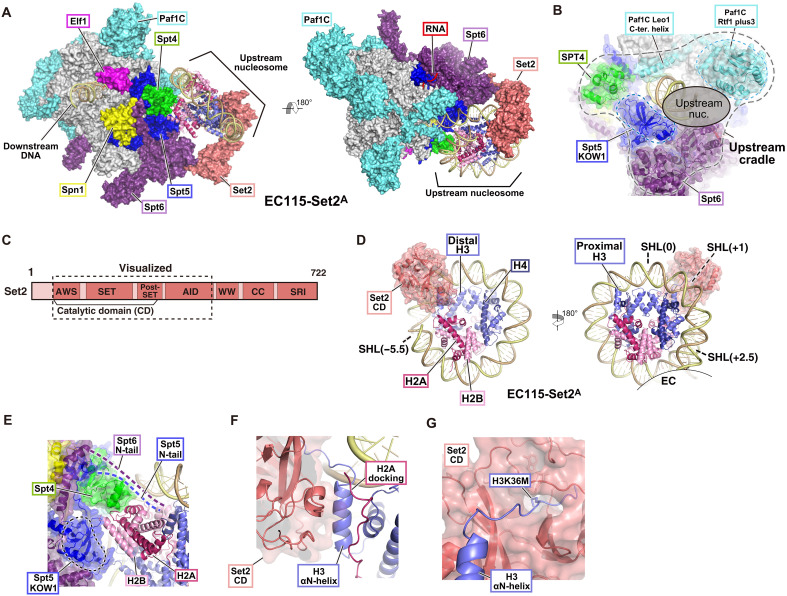
Cryo-EM structure of EC115-Set2. (**A**) Overall structure of EC115-Set2^A^. The EC and Set2 structures are shown in surface models. The nucleosome structure is shown in a ribbon model. (**B**) Close-up view of the upstream surface of the EC. The elongation factors associated with RNAPII are shown in ribbon models with transparent surface models. The nucleosome and Set2 are omitted for clarity. (**C**) Domain structure of the Set2 protein. (**D**) Structure of the nucleosome with the Set2 CD contained in EC115-Set2^A^. The CD is shown in a ribbon model with a transparent surface model. (**E**) Close-up view of the upstream surface of the EC115 contacts. (**F**) Close-up view of the Set2 CD, H3 αN-helix, and H2A docking domain contacts. (**G**) Close-up view of the H3 N-terminal tail and the Set2 CD. The H3 Met36 residue is shown in a stick model.

To understand the mechanism by which Set2 preferentially promotes H3K36me3 deposition as the nucleosome reassembles behind the EC, we performed nucleosome transcription in the presence of Set2 and then analyzed the resulting EC-nucleosome complex species using cryo-EM. Given that H3K36me3 deposition was the most efficient on the EC stalled at the SHL(+6) position within the Temp115 nucleosome (Fig. 1, E and F), we first analyzed the complexes formed on this nucleosome (EC115-Set2 complexes). In this experiment, we used the nucleosome containing H2BK120ub and H3.3K36M, in which the H2BK120 and H3.3K36 residues were replaced by a ubiquitin-conjugated cysteine residue and a methionine residue, respectively (fig. S3, A and B). H2BK120ub reportedly stimulates H3K36me3 deposition by Set2 (*29*), and the H3.3K36M mutant, an oncogenic driver mutation, is known to irreversibly bind the catalytic center of H3K36 methyltransferases (*30*–*34*). To capture snapshot structures during transcription elongation, the EC-nucleosome-Set2 complexes were formed by the in vitro nucleosome transcription reaction and fractionated by sucrose gradient ultracentrifugation in the presence of glutaraldehyde (GraFix). Cryo-EM single-particle analysis yielded two structures, EC115-Set2^A^ and EC115-Set2^B^ (Fig. 2, figs. S3C and S4 to S10, tables S1 and S2, and movie S1).

In the EC115-Set2 structures, we successfully observed Set2 associated with the reassembly intermediate of the nucleosome behind the EC, in which the elongation factors Spt4, Spt5, Spt6, Spn1, Elf1, and Paf1C are visualized together with RNAPII (Fig. 2A and fig. S10A). Spt4, the Spt5 KOW1 domain, Spt6, the Rtf1 Plus3 domain, and the Leo1 C-terminal helix of Paf1C form the “cradle” on the upstream face of the EC, which supports nucleosome reassembly after histone transfer (Fig. 2B) (*20*). Overall, the assembly of the transcription elongation factors on RNAPII is similar between the current EC115-Set2 structures and the previously reported EC115 structure (*20*). The EC115-Set2 structures visualized the Set2 catalytic domain (CD) and AID (Fig. 2C and fig. S10B). Set2 is anchored to Spt6 DLD (death-like domain) through AID, and the Set2 CD binds to the reassembling nucleosome intermediate on the cradle (“on-cradle” structures; Fig. 2, A and D, and fig. S10, A and C).

The two EC115-Set2 complexes accommodate nucleosomes in different orientations with the same Set2 CD-nucleosome interactions, reflecting the flexibility of the Set2 structure. In the EC115-Set2^A^ and EC115-Set2^B^ structures, the Set2 structures differ in the relative orientation between CD and AID because of the flexibility between the two domains (fig. S10B). In addition, the Set2 AID orientation relative to Spt6 differs between the EC115-Set2^A^ and EC115-Set2^B^ structures, indicating that the Spt6-Set2 binding interface also allows flexibility (fig. S10B).

In the EC115-Set2^A^ structure, ~90 bp of DNA [SHL(−5)-SHL(+2.5)] is wrapped around the histone octamer (Fig. 2D). Consistent with our previous report, in the reassembled nucleosome, Spt4 and the Spt5 KOW1 domain directly contact the exposed H2A-H2B and H3-H4 surfaces of the nucleosome on the cradle (Fig. 2E). In addition, a weak cryo-EM density, probably corresponding to the N-terminal tail of Spt5 or Spt6, is observed on the exposed H2A-H2B surface. This finding supports the previous reports that the N-terminal tails of Spt5/Spt6 bind the histone complex and promote nucleosome reassembly (*35*–*37*) (Fig. 2E). In the EC115-Set2^B^ structure, ~80 bp of DNA [SHL(−5)-SHL(+1.5)] is wrapped around the histone octamer (fig. S10C). Although the orientation of the nucleosome relative to RNAPII is different compared to that of the EC115-Set2^A^ structure, the exposed H2A-H2B surfaces contact the EC cradle in both complexes (Fig. 2A and fig. S10A).

In both nucleosome structures, the Set2 CD binds the nucleosomal DNA around the SHL(+1) position and interacts with the H3 αN-helix and H2A C-terminal docking domain, exposed to the solvent resulting from incomplete DNA rewrapping around the SHL(−7)-SHL(−6) region (Fig. 2, D and F, and fig. S10C). The K36M residue in the H3 N-terminal tail is incorporated into the catalytic center of the Set2 CD (Fig. 2G). Thus, Set2 captures the N-terminal tail of one of the H3 copies (promoter-distal H3) within the nucleosome reassembly intermediate for the deposition of H3K36me3. Although this Set2 CD binding to the nucleosome is consistent with the previous studies without RNAPII transcription (*29*, *30*), the ubiquitin moiety of H2BK120C was not visible in the present structures. Consistently, the ubiquitin moiety conjugated to H2B did not affect transcription-coupled H3K36me3 deposition (fig. S11A). We also obtained virtually the same EC115-Set2 complex structures using a nucleosome with no ubiquitin modification (fig. S11, B to G, and table S3).

It should be noted that the position of the reassembled nucleosome is different from that in the previous EC115 structure and is close to the position before transcription (fig. S12). This is probably a consequence of the nucleosome interaction with the Set2 CD, suggesting a chaperoning function of Set2.

### The Set2-EC interaction is essential for transcription-coupled H3K36me3 deposition

**Fig. 3.**
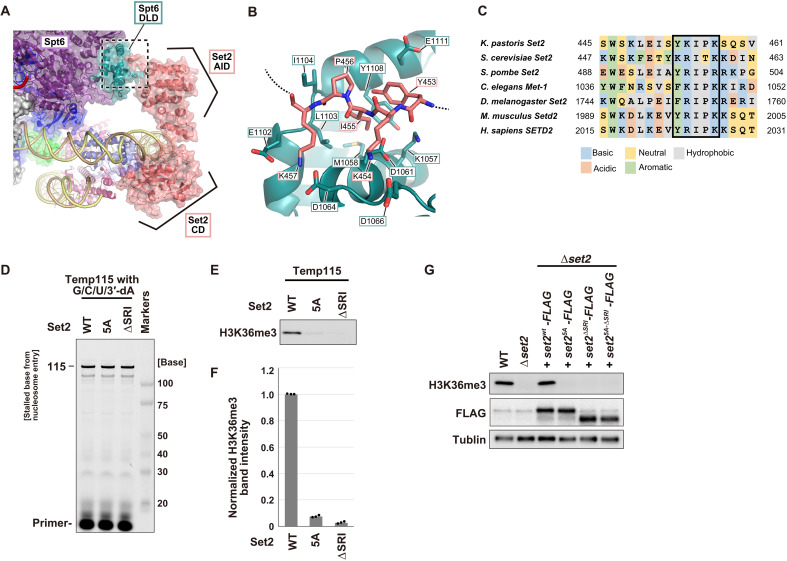
Interface between Set2 and Spt6. (**A**) Close-up view of the Spt6-Set2 upstream nucleosome in EC115-Set2^A^. (**B**) Close-up view of the Spt6-Set2 interaction. This view corresponds to the dotted rectangle in (A). Nitrogen and oxygen are colored blue and red, respectively. The Set2 453-to-457 residues and Spt6 residues, which may interact with Set2, are shown in stick models. (**C**) Sequence alignment of Set2 proteins from various model organisms. The YKIPK motif is enclosed by a black rectangle. (**D**) Urea-PAGE analysis of the elongated RNAs in the transcription assays with the Set2 WT, 5A, and ΔSRI mutants. The transcription reaction was conducted using the Temp115 nucleosome with GTP, CTP, UTP, and 3′-dATP. This experiment was performed in triplicate. (**E**) Western blot of H3K36me3. The samples shown in (D) were analyzed. (**F**) Quantification of the H3K36me3 band intensity shown in (E). The means (bars) and SD (error bars) of the relative values of the H3K36me3 signal intensities compared to that of the Set2 WT in three independent experiments are shown. (**G**) H3K36me3 deposition in yeast *S. cerevisiae* strains carrying the Set2^5A^ and/or Set2^ΔSRI^ mutation, analyzed by Western blotting. Tubulin is a loading control. Three or more yeast strains for each mutant were analyzed, and consistent results were observed.

We found that the region of amino acid residues 453 to 457 (YKIPK) of the Set2 AID directly binds the Spt6 DLD (Fig. 3, A and B). In particular, the Set2 K454 and K457 residues may interact with the acidic region of the Spt6 DLD via electrostatic interactions. In Set2, I455 hydrophobically interacts with the Spt6 M1058, L1103, and Y1108 residues (Fig. 3B). The *K. phaffii* Set2 YKIPK residues in the Spt6-binding motif are mostly conserved in other eukaryotes, including yeast, worm, fly, mouse, and human (Fig. 3C). This Set2 AID-Spt6 interaction tethers the Set2 CD near the upstream reassembling nucleosome and promotes the CD binding to the nucleosome and the H3 N-terminal tail (Fig. 2).

To study the functional relevance of the Set2-Spt6 interaction, we prepared the Set2 mutant Set2^5A^, in which five residues of the Spt6 binding region of Set2 (residues 453 to 457, YKIPK), are replaced by alanine. We then performed the transcription-coupled H3K36me3 deposition assay with the Temp115 nucleosome, which corresponds to an upstream reassembling nucleosome. The Set2^5A^ mutation did not affect the transcription efficiency of the EC with the Temp115 nucleosome (Fig. 3D). The Set2^5A^ mutant is catalytically active, as it is proficient in H3K36me3 deposition under conditions without EC and FACT (fig. S2, D to H). However, the Set2^5A^ mutant was substantially defective in the transcription-coupled H3K36me3 deposition with the Temp115 nucleosome (Fig. 3E, F). These results indicate that the Set2 AID-Spt6 interaction is critical in the transcription-coupled H3K36me3 deposition.

We next tested the H3K36me3 deposition activity of the Set2 SRI deletion mutant (Set2^ΔSRI^), which reportedly has defective binding to the phosphorylated C-terminal tail domain (CTD) of Rpb1, the largest subunit of RNAPII (*26*). Set2^ΔSRI^ reportedly has H3K36me3 deposition activity without EC and FACT (*26*). We found that Set2^ΔSRI^ was markedly defective in transcription-coupled H3K36me3 deposition with the Temp115 nucleosome (Fig. 3, E and F), although its catalytic activity remained intact (fig. S2, D to H). These results suggest that both the Set2 YKIPK motif and Set2 SRI domain are required for efficient H3K36me3 deposition on the nucleosome reassembly intermediate behind the transcribing EC. It should be noted that our pull-down assay revealed that Set2^5A^, Set2^ΔSRI^, and Set2^5A-ΔSRI^ were associated with the EC (fig. S13), suggesting the existence of another EC binding site in Set2.

**Fig. 4.**
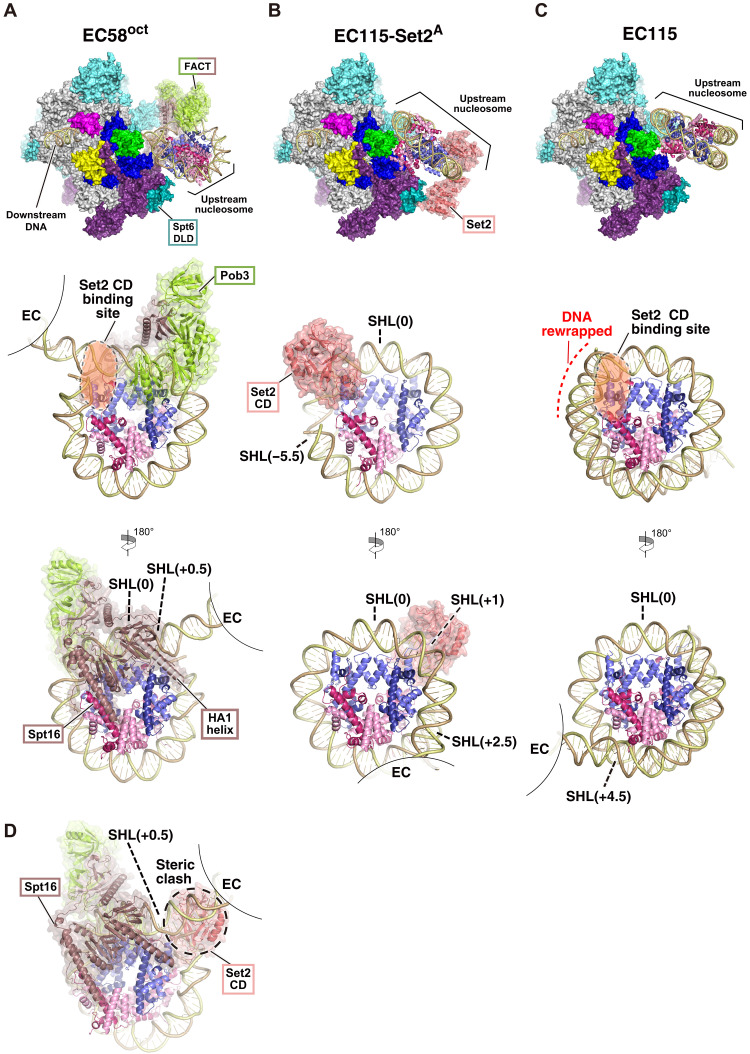
Structural comparison of the reassembling nucleosomes. (**A** to **C**) Overall and nucleosome structures contained in EC58^oct^ (A), EC115-Set2^A^ (B), and EC115 (C). The FACT and Set2 CD structures are shown in ribbon models with a transparent surface model. The HA1 helix of FACT Spt16 is highlighted. (**D**) Superimposed view of the nucleosome-FACT of EC58^oct^ with the Set2 CD of EC115-Set2^A^. The Set2 CD clashes with the unwrapped nucleosomal DNA SHL(+1) of EC58^oct^.

To test the effects of the Set2^5A^ and Set2^ΔSRI^ mutations in cells, we constructed yeast *Saccharomyces cerevisiae* strains carrying either the Set2^5A^, Set2^ΔSRI^, or Set2^5A-ΔSRI^ mutation (table S4). The Set2^5A^, Set2^ΔSRI^, and Set2^5A-ΔSRI^ cells were profoundly defective in H3K36me3 deposition (Fig. 4G). Defective H3K36me3 deposition in the Set2^ΔSRI^ mutant is consistent with previous reports (*25*, *26*). Therefore, both the Set2 AID-Spt6 interaction and Set2 SRI-RNAPII CTD interaction play essential roles in H3K36me3 deposition in cells.

### Set2 CD binding is incompatible with FACT binding on the reassembling nucleosome

Previously, we reported that the EC stalled at the position 58 bp from the nucleosome entry (EC58^oct^) contains a subnucleosome structure on the cradle, in which ~75 bp of DNA [SHL(−7)-SHL(+0.5)] is wrapped around the histone octamer complexed with FACT (*20*). In EC58^oct^, FACT contacts the nucleosomal DNA SHL(−1)-SHL(0) region, and the HA1 helix of the FACT Spt16 subunit binds the exposed surface of H3-H4 corresponding to the SHL(+1) region, instead of DNA (Fig. 4A). Superimposition of the Set2 part of the EC115-Set2 complex on the EC58^oct^ revealed the mutual exclusivity of Set2 and FACT binding on the reassembling nucleosome, because the Set2 CD would cause a steric clash with the DNA around SHL(+1) in the subnucleosome intermediate complexed with FACT (Fig. 4D). Given that the Set2 CD binds the nucleosome in which the nucleosomal DNA SHL(+1) is rewrapped (Fig. 4B), the Set2 CD may be able to bind the nucleosome after FACT dissociates from the H3-H4 surface corresponding to the SHL(+1) DNA region.

Consistently, further classification identified an EC class that is simultaneously associated with both FACT and Set2 (the EC115^hex^-Set2-FACT complex) (fig. S7, table S2, and movie S1). This complex harbors a hexasome, a reassembling nucleosome intermediate containing one H2A-H2B and two H3-H4, on the upstream side of the EC, where FACT is tightly bound to the nucleosome^hex^ as in the previous structure (EC58^hex^) (*20*). While Set2 interacts with the EC through the Set2 AID-Spt6 DLD interaction, the Set2 CD is invisible on the nucleosome^hex^ (fig. S7B). Thus, although Set2 and FACT can bind simultaneously to the EC, the Set2 CD will only deposit H3K36me3 after FACT dissociates from the nucleosome.

We also compared the current EC115-Set2 structures with the previously reported EC115 structure (*20*). In EC115, ~120 bp of DNA [SHL(−7)-SHL(+4)] is rewrapped around the histone octamer (Fig. 4C). The rewrapped nucleosomal DNA end around the SHL(−7)-SHL(−6) region conceals the H3 αN-helix. In contrast, in the EC115-Set2 complexes, the SHL(−7)-SHL(−6) region is stripped from the histone surfaces and the Set2 CD contacts the exposed H3 αN-helix (Fig. 4B). Therefore, the nucleosome in EC115 is incompatible with Set2 CD binding. This suggests that Set2 may need to complete H3K36me3 deposition before rewrapping the SHL(−7)-SHL(−6) region, although we cannot exclude the possibility that Set2 can actively unwrap this region, as reported in previous studies (*29*, *30*).

### Structures of the EC130-Set2 complexes (“off-cradle” structures)

**Fig. 5.**
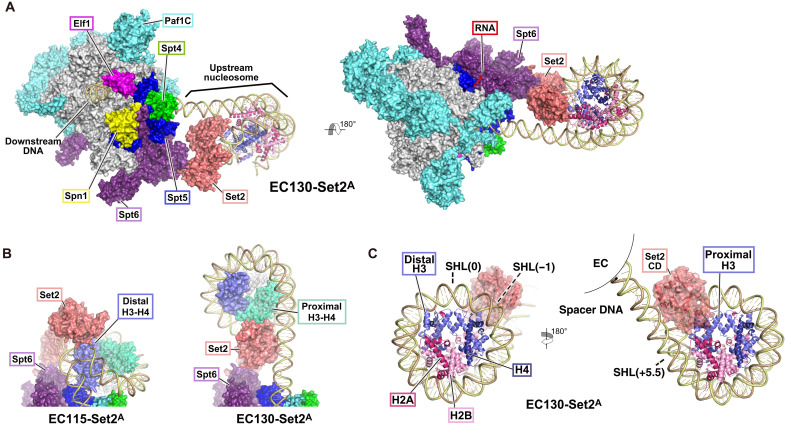
Cryo-EM structure of EC130-Set2. (**A**) Overall structure of EC130-Set2^A^. The EC and Set2 structures are shown in surface models. The nucleosome structure is shown in a ribbon model. (**B**) Comparison of the Set2-histone interactions between EC115 and EC130. The promoter-distal H3-H4 and promoter-proximal H3-H4 in the DNA template are colored blue and cyan, respectively. Rtf1, a Paf1C subunit, is omitted for clarity. (**C**) Nucleosome-Set2 CD structures contained in EC-Set2^A^. The CD is shown in a ribbon model with a transparent surface model.

The EC115-Set2 complexes represent the state where the Set2 CD captures the N-terminal tail of the promoter-distal copy of H3 for trimethylation. To address whether Set2 can methylate the other promoter-proximal copy of H3, we determined the cryo-EM structures of the EC130-Set2 complexes, in which the EC had advanced by 15 bp (~1.5 helical pitch of DNA) on the Temp130 nucleosome (figs. S14 to S16, tables S5 and S6, and movie S1). Given that H2B ubiquitination is not crucial for transcription-coupled H3K36me3 deposition, we omitted it in this experiment. We obtained two structures, EC130-Set2^A^ and EC130-Set2^B^ (Fig. 5A and fig. S17A), in which the Set2 CD captures the opposite face of the nucleosome to methylate the promoter-proximal H3 (Fig. 5B and fig. S17B).

In contrast to the EC115-Set2 complexes, spacer DNA segments comprising 24 and 15 bp are observed between the RNAPII DNA-exit tunnel and the nucleosome entry site in the EC130-Set2^A^ and EC130-Set2^B^ complexes, respectively (fig. S17C). Therefore, the reassembled nucleosomes in the EC130-Set2 complexes do not directly contact the EC cradle (“off-cradle” structures) (Fig. 5A and fig. S17A). The absence of the nucleosome-cradle interaction may render the reassembled nucleosome more flexible.

In the EC130-Set2^A^ complex, the spacer DNA is straight, supported by the C-terminal helix of Leo1, a subunit of Paf1C (fig. S17D). In contrast, the spacer DNA of the EC130-Set2^B^ complex is sharply kinked at the RNAPII DNA-exit site, and the Leo1 C-terminal helix is disordered. This suggests that Leo1 may function to support the nucleosome position relative to the EC. In the EC130-Set2 complexes, the Set2 AID is anchored to the Spt6 DLD, as observed in the EC115-Set2 complexes. However, Set2 intervenes between Spt6 and the nucleosome by adopting a twisted conformation (fig. S17E). This change is achieved through a flexible hinge between the Set2 CD and the Set2 AID.

In these complexes, ~130 bp of DNA [approximately SHL(−7)-SHL(+5.5)] is wrapped around the histone octamer (Fig. 5C and fig. S17F). The Set2 CD interacts with the H3 αN-helix, the H2A C-terminal docking domain, and the spacer DNA to capture the promoter-proximal H3 tail, which is in stark contrast with the Set2 CD binding to the promoter-distal H3 tail in the EC115-Set2 complexes. This suggests that Set2 can catalyze H3K36me3 on both of the H3 tails as the EC traverses the nucleosome.

## DISCUSSION

In the present study, we demonstrated that nucleosomal H3K36me3 deposition by Set2 requires transcription elongation through the nucleosome by the EC. The cryo-EM structures of the EC-nucleosome-Set2 complexes revealed that Set2 is tethered to Spt6, becoming an integral component of the EC. The structures capture the H3K36me3 deposition states during nucleosome reassembly and reveal the crucial interactions between Set2, Spt6, and the reassembling nucleosome intermediate. We observed two modes of Set2 interactions. Set2 flexibly changes the CD and AID orientations, allowing the CD to capture the reassembling nucleosome intermediate either at the nucleosome cradle of the EC (on-cradle, EC115) or far from the cradle (off-cradle, EC130). This flexibility may enable H3K36me3 deposition on both copies of H3 within the nucleosome according to the EC progression.

We found that H3K36me3 deposition by Set2 is substantially enhanced when the EC passes through the SHL(0) position of the nucleosome. This finding suggests that Set2 primarily promotes H3K36me3 deposition during the nucleosome reassembly process on the upstream side of the EC. In the EC-nucleosome-Set2 structures, we found that the Set2 AID binds to the Spt6 subunit of the transcribing EC via the Spt6-binding motif (YKIPK), which is conserved from yeast to human (Fig. 3C). Our mutational analysis revealed that the interaction between the Set2 AID (the YKIPK motif) and the Spt6 DLD is essential for transcription-coupled H3K36me3 deposition (Fig. 3, E to G). The Set2-Spt6 interaction may optimize the positioning of the Set2 CD to interact with the nucleosomal DNA and the H3 αN-helix region, facilitating efficient H3K36me3 deposition during nucleosome reassembly by the EC. SETD2, the human counterpart of Set2, is implicated in tumorigenesis. SETD2 mutations and loss of function have been found in various tumors (*38*, *39*). Notably, they include mutations in the conserved SPT6 interaction motif (^2023^YRIPK^2027^) and, in particular, the R2024Q mutation (fig. S18). These findings underscore the critical role of the Spt6/SPT6-Set2/SETD2 interaction in H3K36me3 deposition and gene regulation. Furthermore, a mutation of the RNAPII-binding region (SRI) also impaired transcription-coupled H3K36me3 deposition (Fig. 3, E to G), highlighting the importance of the interactions between the Set2 SRI domain and the RNAPII CTD.

We found that in the EC115-Set2 complexes, ~80 to 90 bp of DNA is rewrapped (Fig. 4B). This is an intermediate rewrapping state between the previous EC58 (60 bp) and EC115 (120 bp) structures (Fig. 4, A to C). This may suggest that Set2 begins the deposition of H3K36me3 during the transition from EC58 to EC115. Our current structure of the EC115^hex^-Set2-FACT complex shows that both Set2 and FACT can bind simultaneously to the EC. However, the Set2 CD and FACT cannot bind to the nucleosome simultaneously. The Set2 CD is likely to become accessible to the nucleosome upon FACT dissociation from the nucleosome.

**Fig. 6.**
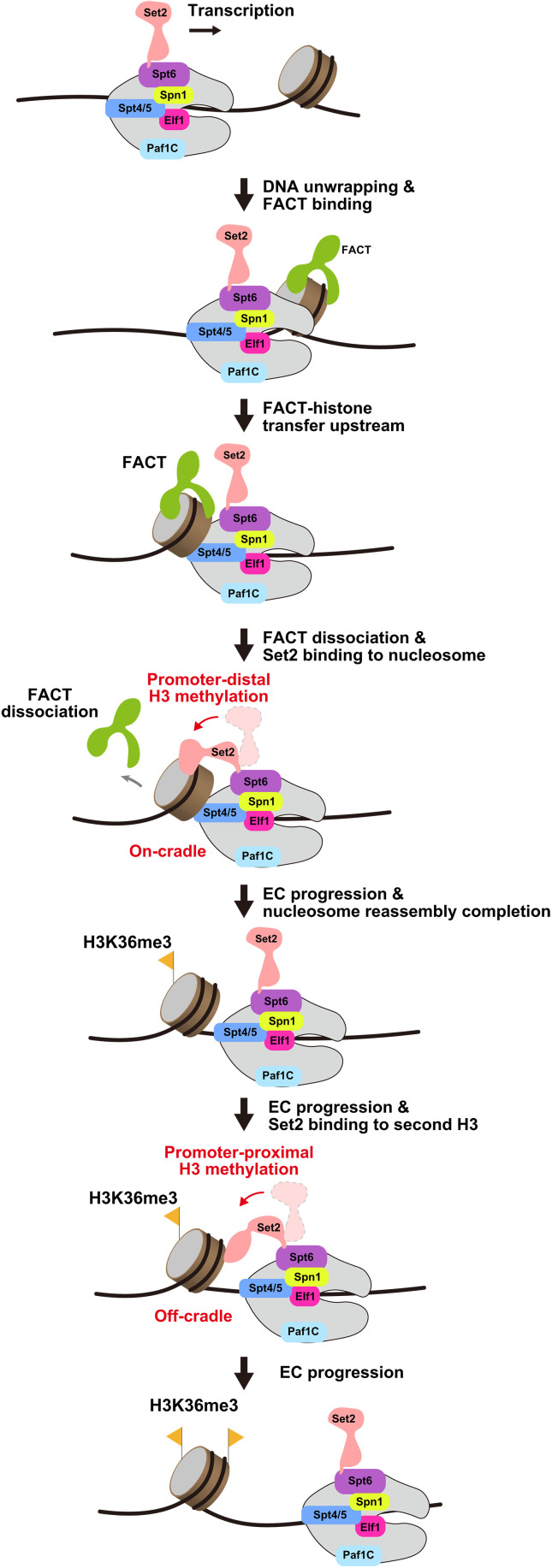
Model of H3K36me3 deposition during transcription through the nucleosome. See Discussion.

On the basis of these results, we propose a model of transcription-coupled H3K36me3 deposition (Fig. 6). Set2 is an integral part of the EC, accompanying transcription elongation in gene bodies. When the EC passes through a nucleosome, histones are transferred from the downstream side of the EC to the upstream side, where nucleosome reassembly begins (top, second, and third rows). In the early stage of the reassembly, the histones in the reassembling nucleosome intermediate are bound by FACT and the DNA is still unwrapped to SHL(+1), which are both incompatible with the Set2 CD binding to the nucleosome (third row). Therefore, while Set2 is tethered to Spt6 through the AID, the Set2 CD is unable to bind to the nucleosome and is in a “standby” mode (third row). As the EC advances, the DNA would gradually rewrap the histone surfaces, facilitating FACT detachment from the histones (fourth row). When the DNA is rewrapped around SHL(+1), the Set2 CD binds to the nucleosome to catalyze H3K36me3 deposition on the tail of the promoter-distal copy of H3 (fourth row). After the reaction, the Set2 CD probably dissociates from the SHL(+1) site of the nucleosome, and further nucleosome rewrapping proceeds (fifth row). The Set2 CD binds to the opposite side of the nucleosome at SHL(−1) and deposits H3K36me3 on the other copy of H3 (sixth row). Thus, the nucleosome with double H3K36me3 marks is generated after the passage of the EC (bottom rows). It should be noted that the two H3K36me3 marks are not necessarily equally introduced, as methylation is more efficient on the on-cradle complex than the off-cradle complex. The trapping of the reassembling nucleosome on the cradle may render the nucleosome position favorable for H3K36me3 deposition by Set2.

Our present study highlights the important role of FACT in regulating H3K36me3 deposition. In the absence of FACT, Set2 promotes H3K36me3 deposition in both the upstream and downstream nucleosomes (Fig. 1, G and H). As the Spt6-tethered Set2 is located far from the downstream nucleosome, H3K36me3 deposition in the downstream nucleosome likely involves a different molecule of Set2 in trans. FACT may inhibit such a trans reaction, ensuring the coupling between H3K36me3 and transcription. This also suggests that FACT suppresses aberrant H3K36me3 deposition in the genome. Previous genetic analyses showed that temperature-sensitive yeast FACT mutants exhibit H3K36me3 mislocalization and substantial growth defects (*25*, *40*). In the absence of FACT, Set2 would promiscuously deposit H3K36me3 on inappropriate genomic regions, consequently disturbing cell viability. Thus, FACT is a critical factor for the strict coordination of H3K36me3 deposition with transcription, thereby preventing abnormal gene activation in chromatin.

Recently, the cryo-EM structures of mammalian ECs bound with SETD2 and an upstream/downstream nucleosome were reported (*36*). The downstream nucleosome complex contained SETD2 bound to the nucleosome, probably because this complex was reconstituted in the absence of FACT. The upstream nucleosome complexes (20- and 30-bp complexes) were prepared by placing the EC downstream of a preexisting nucleosome (with no transcription). The upstream nucleosomes showed an off-cradle arrangement, with longer spacer DNA segments (26 and 35 bp between the EC cradle and the nucleosome) as compared with the current EC130 structures yielded by nucleosome transcription (15 and 24 bp; fig. S19). In these mammalian upstream complexes, the nucleosome is unwrapped to SHL(+4), and the SETD2 CD does not contact the spacer DNA, in contrast to our yeast structures. Although a low-resolution (15.8 Å) map (+115 complex) yielded by transcription in the presence of FACT was also reported, the SETD2 arrangement is off-cradle and different from the on-cradle arrangement in our EC115-Set2 structure. These differences could arise from the design of the reconstituted mammalian complexes or species-specific differences. Further examination is required to clarify these points.

In this study, we obtained structural snapshots of transcription-coupled H3K36me3 deposition by analyzing the complexes formed during transcription elongation, including the nucleosome reassembly process. These structures offer a valuable framework for understanding how active histone marks are spread across transcribed gene bodies.

## MATERIALS AND METHODS

### Protein preparation

*K. phaffii* RNAPII, Spt4/5, Elf1, Spn1, Spt6, Paf1C, FACT, human positive transcription elongation factor b (P-TEFb), and human histones H2A, H2B [wild type (WT) and K120C mutant], H3.1, H3.3 (WT and K36M mutant), and H4 were purified as described previously (*20*, *41*–*43*). The histone octamers, except for that containing H2BK120Cub, were prepared as described previously (*43*).

H2BK120Cub, in which ubiquitin is conjugated to Cys^120^ of the H2B protein, was prepared as described previously with modifications (*44*). Hexahistidine (His6)–tagged ubiquitin was purified as described previously. The H2BK120C and His6-ubiquitin powders were separately dissolved to 10 mg/ml in denaturing buffer adjusted to pH 8.5 (50 mM sodium tetraborate and 6 M urea). The resulting protein solutions were mixed in a 1:1 molar ratio, and then 5 mM tris(2-carboxyethyl)phosphine (TCEP) was added. After rotation at room temperature for 30 min, the sample was cooled on ice. Then, 0.44 mM DCA (1,3-dichloroacetone) dissolved in DMF (*N*,*N*-dimethylformamide) was added to cross-link the histone and ubiquitin, and the solution was incubated on ice for 30 min. Afterward, 5 mM 2-mercaptoethanol was added to quench the cross-linking reaction. The cross-linked sample was dialyzed against Ni wash buffer [50 mM tris-HCl (pH 8.0), 500 mM NaCl, 6 M urea, 5% glycerol, and 5 mM imidazole]. The dialyzed sample was purified using Ni-NTA column chromatography and eluted by Ni wash buffer containing 500 mM imidazole. The resulting sample contains a ubiquitin monomer, ubiquitin dimer, and H2B-ubiquitin. This sample was dialyzed against MonoS wash buffer [20 mM KOAc (pH 5.2), 200 mM NaCl, 6 M urea, 1 mM EDTA, and 5 mM 2-mercaptoethanol] and purified by MonoS cation exchange column chromatography, with elution by MonoS wash buffer containing 900 mM NaCl. The fractions containing H2B-ubiquitin were collected. The purified H2B-ubiquitin was dialyzed against distilled water and stored at −80°C. For the preparation of the histone octamer containing H2BK120ub, H2A, H2BK120Cub, H3.3, and H4 were dissolved in denaturing buffer [20 mM tris-HCl (pH 7.5), 7 M guanidine-HCl, and 20 mM 2-mercaptoethanol] to a total protein concentration of 1.0 mg/ml. The sample was refolded during dialysis against refolding buffer [10 mM tris-HCl (pH 7.5), 2 M NaCl, 1 mM EDTA, and 5 mM 2-mercaptoethanol], purified by Superdex200 gel filtration chromatography, flash-frozen in liquid nitrogen, and stored at −80°C.

Mouse CK2 was purified as described previously with modifications (*45*). The DNA fragments encoding the CK2α and CK2β subunits were inserted into the pRSFDuet vector. The CK2α and CK2β subunits were coexpressed as recombinant proteins in *Escherichia coli* BL21 (DE3) RIL cells. The His6 tag was fused to the CK2α subunit. The cultured cells were harvested by centrifugation, suspended in lysis buffer [50 mM tris-HCl (pH 7.5), 500 mM NaCl, 1 mM phenylmethylsulfonyl fluoride (PMSF), and 20 mM imidazole], and disrupted by sonication. The disrupted cells were centrifuged, and the His6-tagged CK2α-CK2β proteins contained in the supernatant were purified by Ni-NTA column chromatography. The Ni beads were washed with lysis buffer, followed by an additional wash with Ni wash buffer [50 mM tris-HCl (pH 7.5), 100 mM NaCl, 1 mM PMSF, and 20 mM imidazole]. The CK2α-CK2β protein was eluted with Ni wash buffer containing 300 mM imidazole. The resulting protein was purified by HiTrap Heparin chromatography (Cytiva) using heparin wash buffer [20 mM tris-HCl (pH 7.5), 100 mM NaCl, 1 mM EDTA, and 2 mM 2-mercaptoethanol] and eluted with heparin wash buffer containing 1 M NaCl. The resulting protein was further purified on a Superose 6 Increase gel filtration column using size exclusion chromatography buffer [20 mM tris-HCl (pH 7.5) and 1 M NaCl]. The fractions containing the CK2α protein or CK2α-CK2β complex were separately collected. The CK2α protein was dialyzed against dialysis buffer [20 mM Hepes-NaOH (pH 7.5), 300 mM NaCl, 0.5 mM EDTA, 5% glycerol, and 1 mM dithiothreitol (DTT)], flash-frozen in liquid nitrogen, and stored at −80°C.

*K. phaffii* Set2 for the cryo-EM analysis was expressed as an HRV-3C cleavable, N-terminally His6-tagged protein in *E. coli*. The cells were first resuspended in buffer A [20 mM tris-HCl (pH 8.0), 700 mM NaCl, and 10 mM 2-mercaptoethanol] supplemented with 0.1 mM PMSF and then disrupted by sonication. The lysate was cleared by centrifugation and applied to a Ni Sepharose 6 Fast Flow column (Cytiva). The column was washed serially with buffer A supplemented with 20 mM imidazole and buffer B150-20 [20 mM tris-HCl (pH 8.0), 150 mM NaCl, 10 mM 2-mercaptoethanol, and 20 mM imidazole]. The column was treated overnight with HRV-3C protease to cleave the tag. The protein was eluted with buffer B150-20 and then diluted twofold using buffer C [20 mM Hepes-KOH (pH 7.5), 0.1 μM zinc acetate, 0.1 mM TCEP-HCl (TCEP hydrochloride), and 5% glycerol]. The protein was further purified by Resource Q anion-exchange column chromatography (Cytiva) using a linear gradient of buffer C to buffer D [20 mM Hepes-KOH (pH 7.5) and 2 M potassium acetate]. Fractions containing Set2 were collected, then concentrated, and buffer exchanged to buffer E [20 mM Hepes-KOH (pH 7.5), 150 mM potassium acetate, 0.1 μM zinc acetate, 0.1 mM TCEP-HCl, and 5% glycerol] using an Amicon Ultra filter (Millipore). *K. phaffii* Set2 and its mutant for the assays were also expressed as HRV-3C cleavable, N-terminally His6-tagged proteins in *E. coli* and purified using an improved protocol with better yields. The cells were resuspended in buffer A and disrupted by sonication. The lysate was cleared by centrifugation and applied to a Ni Sepharose 6 Fast Flow column (Cytiva). The column was washed serially with buffer A supplemented with 20 mM imidazole and buffer B300-20 [20 mM tris-HCl (pH 8.0), 300 mM NaCl, 10 mM 2-mercaptoethanol, and 20 mM imidazole] and then treated overnight with HRV-3C protease. The protein was eluted with buffer B300-20, diluted twofold using buffer C, and then further purified by Resource S cation-exchange column chromatography (Cytiva) using a linear gradient of buffer C to buffer D. For the ΔSRI variants, Resource Q anion-exchange column chromatography (Cytiva) was carried out instead of Resource S. Fractions containing Set2 were concentrated and buffer exchanged to buffer E using an Amicon Ultra filter (Millipore).

The His-tagged Set2 and its mutants for assays were expressed similarly to the tag-cleaved versions mentioned above. After sample application to a Ni Sepharose 6 Fast Flow column (Cytiva), the column was washed serially with buffer A supplemented with 30 mM imidazole and buffer B300-30 [20 mM tris-HCl (pH 8.0), 300 mM NaCl, 10 mM 2-mercaptoethanol, and 30 mM imidazole]. The protein was eluted with buffer B150-250 [20 mM tris-HCl (pH 8.0), 150 mM NaCl, 10 mM 2-mercaptoethanol, and 250 mM imidazole] and then dialyzed against buffer B300 [20 mM tris-HCl (pH 8.0), 150 mM NaCl, and 10 mM 2-mercaptoethanol]. Afterward, the proteins were further purified by ion-exchange column chromatography and then concentrated in buffer E, similarly to the tag-cleaved versions.

### Preparation of template DNAs

All nucleosomal template DNA sequences were designed on the basis of the Widom601 DNA sequence (*46*). The Temp49 and Temp115 DNA fragments were designed and purified as described previously (*20*). The DNA sequences of Temp42 and Temp58 were designed as described below. Briefly, the DNA fragments were amplified by polymerase chain reaction, cleaved by *Bgl* I, and purified by nondenaturing polyacrylamide gel electrophoresis (PAGE) using a Prep Cell (Bio-Rad) or Superose 6 Increase gel-filtration column chromatography (Cytiva). The purified DNA fragments were concentrated by an Amicon Ultra 3K centrifugal concentrator (Millipore) and stored at −20°C. The DNA sequences of Temp42, Temp58, Temp130, and Temp140 are as follows: Temp42: nontemplate strand, 5′-TGGCCGTTTTCGTTGTTTTTTTCTGTCTCGTGCCTGGTGTCTTGGGTGTAAAACCCTTGGCGGTTAAAACGCGGGGGACAGCGCGTACGTGCGTTTAAGCGGTGCTAGAGCTGTCTACGACCAATTGAGCGGCCTCGGCACCGGGATTCTGAT-3′; template strand, 5′-ATCAGAATCCCGGTGCCGAGGCCGCTCAATTGGTCGTAGACAGCTCTAGCACCGCTTAAACGCACGTACGCGCTGTCCCCCGCGTTTTAACCGCCAAGGGTTTTACACCCA-AGACACCAGGCACGAGACAGAAAAAAACAACGAAAACGGCCACCA-3′; Temp58: nontemplate strand, 5′-TGGCCGTTTTCGTTGTTTTTTTCTGTCTCGTGCCTGGTGTCTTGGGTGTTTTCCCCTTGGCGGTTAAAACGCGGGGGACAGCGCGTACGTGCGTTTAAGCGGTGCTAGAGCTGTCTACGACCAATTGAGCGGCCTCGGCACCGGGATTCTGAT-3′; template strand, 5′-ATCAGAATCCCGGTGCCGAGGCCGCTCAATTGGTCGTAGACAGCTCTAGCACCGCTTAAACGCACGTACGCGCTGTCCCCCGCGTTTTAACCGCCAAGGGGAAAACACCC-AAGACACCAGGCACGAGACAGAAAAAAACAACGAAAACGGCCACCA-3′; Temp130: nontemplate strand, 5′-TGGCCGTTTTCGTTGTTTTTTTCTGTCTCGTGCCTGGTGTCTTGGGTG-TTTTCCCCTTGGCGGTTTTTTCGCGGGGGTCTGCGCGTTCGTGCGTTTTTGCGGTGCTTGTGCTGTCTTCGT-CCTTTTGTGCGGCCTCGAAAACGGGATTCTGAT-3′; template strand, 5′-ATCAGAATCCCGTTTTCGAGGCCGCACAAAAGGACGAAGACAGCACAAGCACCGCAAAAACGCACGAACGCGCAGACCCCCGCGAAAAAACCGCCAAGGGGAAAAC-ACCCAAGACACCAGGCACGAGACAGAAAAAAACAACGAAAACGGCCACCA-3′; Temp140: nontemplate strand, 5′-TGGCCGTTTTCGTTGTTTTTTTCTGTCTCGTGCCTGGTGTC-TTGGGTGTTTTCCCCTTGGCGGTTTTTTCGCGGGGGTCTGCGCGTTCGTGCGTTTTTGCGGTGCTTGTGCTGTCTTCGTCCTTTTGTGCGGCCTCGGCTCCGGGTTAAAAAT-3′; template strand, 5′-ATTTTTAACCCGGAGCCGAGGCCGCACAAAAGGACGAAGACAGCACAAGCACCGCAAAAACGCAC-GAACGCGCAGACCCCCGCGAAAAAACCGCCAAGGGG-AAAACACCCAAGACACCAGGCACGAGACAGAAAAAAACAACGAAAACGGCCACCA-3′.

### Nucleosome preparation

The template nucleosomes containing H2A, H2B (WT or K120Cub), H3.3 (WT or K36M mutant), and H4 were reconstituted by the salt dialysis method and purified as described previously (*43*). After dialysis, the 45-bp short double-stranded DNA fragment (*17*) was ligated to the sticky end of the nucleosomal DNA. For the nucleosome containing H2BK120Cub, the His6 tag fused to ubiquitin was cleaved by tobacco etch virus protease. The resulting nucleosomes were then purified by nondenaturing PAGE using a Prep Cell (Bio-Rad). The nucleosome containing H2A, H2B, H3.1, H4, and the Widom601 193-bp DNA (*47*) was prepared as described previously (*20*, *43*). The purified nucleosomes were flash-frozen in liquid nitrogen and storedat −80°C.

### Transcription-coupled H3K36me3 deposition assay

For the transcription-coupled H3K36me3 deposition assay with EC stalling, the transcription reaction was performed by mixing the indicated template nucleosomes, RNAPII, TFIIS, Spt4/5, Elf1, Paf1C, Spt6, Spn1, FACT, P-TEFb, CK2α, Set2, and a DY647 fluorescently labeled RNA primer (Dharmacon) in 30 μl of reaction solution containing a uridine 5′-triphosphate (UTP) and 3′-dATP combination or a UTP, GTP, CTP, and 3′-dATP combination in the presence of SAM. The resulting reaction mixture was incubated at 37°C for 1 hour. The RNAPII EC proceeded and stalled at the first C position with the UTP and 3′-dATP combination or the first T position with the UTP, GTP, CTP, and 3′-dATP combination. CK2 phosphorylates Spt5, Spt6, and FACT, promoting Spt6 binding to Spn1 and FACT binding to histones (*48*–*51*). P-TEFb phosphorylates the RNAPII C-terminal domain, Spt5, and Paf1C, promoting the EC assembly (*52*, *53*). The resulting reaction mixture contained 0.1 μM template nucleosome, 0.1 μM RNAPII, 0.1 μM TFIIS, 0.4 μM Spt4/5, 1.0 μM Elf1, 0.2 μM RNA, 0.27 μM CK2α, 0.1 μM P-TEFb, 0.4 μM Spt6, 0.5 μM Spn1, 0.4 μM Paf1C, 0.5 μM FACT, and 0.1 μM Set2 in 31 mM Hepes-KOH (pH 7.5) buffer containing 68 mM KOAc, 0.4 μM Zn(OAc)_2_, 0.04 mM TCEP, 2.5% glycerol, 15 mM NaCl, 0.2 mM DTT, 5 mM MgCl_2_, 0.02 mM EDTA, 0.1 mM SAM, and NTPs (nucleoside triphosphates; 0.4 mM UTP and 0.4 mM 3′-dATP or 0.4 mM UTP, 0.4 mM GTP, 0.4 mM CTP, and 0.4 mM 3′-dATP).

For the transcription-coupled H3K36me3 deposition assay without EC stalling in Fig. 1C, the Temp42 nucleosome was used as the template. The transcription reaction was conducted as described above using ATP instead of 3′-dATP.

For the H3K36me3 deposition assay with or without the EC in fig. S2B, the transcription reaction was conducted as described above with or without EC components, RNAPII, TFIIS, Spt4/5, Elf1, Paf1C, Spt6, Spn1, and P-TEFb. For the transcription-coupled H3K36me3 deposition assay with or without the ubiquitination of H2BK120C in fig. S11A, the transcription reaction was conducted as described above using 1.0 μM Set2 and the Temp115 nucleosome with or without H2BK120Cub.

For the urea-PAGE analysis of RNA, 1 μl of the reaction mixture was mixed with 0.5 μl of stop solution [100 mM tris-HCl (pH 7.5), proteinase K (1.0 mg/ml; Roche), 150 mM EDTA, and 4 M urea] and incubated at room temperature for 10 min, followed by addition of 5 μl of HiDi formamide (Thermo Fisher Scientific) and heating at 95°C. The resulting RNA was analyzed by 10% denaturing urea-PAGE. Blue-colored RNA markers (Biodynamics Laboratory) were used. The DY647 fluorescent signal was detected by an Amersham Typhoon imager (Cytiva) through a glass plate. The images were adjusted by ImageJ (*54*) with linear contrast enhancement.

For the Western blot analysis of H3K36me3, 12 μl of the reaction mixture was mixed with 4 μl of 4× SDS buffer and then heated at 95°C. A 15-μl portion of the resulting sample was fractionated by SDS-PAGE (Nacalai Tesque, Extra PAGE One Precast Gel 10 to 20%, cat. no. 13068-24). PageRuler Plus (Thermo Fisher Scientific, cat. no. 26619) protein markers were used. After electrophoresis, the proteins in the gel were transferred to a membrane (Amersham Protran Premium NC 0.2 μm, cat. no. 10600009). The membrane was blocked with Blocking One (Nacalai Tesque, cat. no. 03953-66) for 20 min. After washing with tris-buffered saline with Tween 20 (TBS-T), the membrane was incubated with a mouse monoclonal anti-H3K36me3 antibody (Active Motif, cat. no. 61022, 1:1000) overnight at 4°C. The antibody was diluted with Can Get Signal Solution 1 (TOYOBO, cat. no. NKB-201). After the membrane was washed with TBS-T, it was incubated with a goat anti-mouse immunoglobulin G (IgG) conjugated with Cy3 (Jackson Immuno Research Laboratories Inc., cat. no. 115-165-146, 1:1000) for 1 hour. This antibody was diluted with Can Get Signal Solution 2 (TOYOBO, cat. no. NKB-301). After washing the membrane with TBS-T, the fluorescence signals of the membrane were detected by an Amersham Typhoon imager. The band signal intensities corresponding to H3K36me3 were quantitated by ImageJ (*54*), and the relative signal intensities compared to the indicated sample were calculated. The mean and SD values of three or four independent experiments were calculated and plotted.

### H3K36me3 deposition assay without transcription

Histone methylation was performed by mixing the nucleosome containing the Widom601 193-bp DNA (*47*), Set2, SAM and a DY647 fluorescently labeled RNA primer (Dharmacon) in 30 μl of reaction solution and incubating the mixture at 37°C for 1 hour. The resulting reaction mixture contains the 0.1 μM template nucleosome, 0.2 μM RNA, and 0.1 μM Set2 in 31 mM Hepes-KOH (pH 7.5) buffer containing 68 mM KOAc, 0.4 μM Zn(OAc)_2_, 0.04 mM TCEP, 2.5% glycerol, 15 mM NaCl, 0.2 mM DTT, 5 mM MgCl_2_, 0.02 mM EDTA, 0.1 mM SAM, 0.4 mM UTP, 0.4 mM GTP, 0.4 mM CTP, and 0.4 mM 3′-dATP. A 13.5-μl portion of the reaction was mixed with 4.5 μl of 4× SDS buffer and heated at 95°C for 1 min. The resulting samples were analyzed by Western blotting, and the H3K36me3 signal was detected as described in the “Transcription-coupled H3K36me3 deposition assay” section.

### Set2 pull-down assay

The EC was reconstituted as described in the “Transcription-coupled H3K36me3 deposition assay” section using 1.0 μM His-tagged Set2. The 45-bp double-stranded DNA fragment (*17*) was used as the template, instead of the template nucleosome. A 30-μl portion of the resulting reaction mixture was mixed with anti-FLAG antibody beads (Sigma-Aldrich, cat. no. A2220), which were preblocked by bovine serum albumin, and rotated for 3 hours at 4°C. During the incubation, the His-FLAG–tagged Rpb2, a subunit of RNAPII, was bound to the beads. The reaction mixtures without RNAPII were also prepared for a negative control. After washing the beads with wash buffer [20 mM tris-HCl (pH 7.5), 100 mM NaCl, and 0.1% NP-40], the tagged RNAPII was eluted with 30 μl of wash buffer containing the FLAG peptide (0.5 mg/ml). Aliquots (15 μl) of the eluted samples were mixed with 5 μl of 4× SDS buffer and then heated at 95°C. Western blotting was conducted as described in the “Transcription-coupled H3K36me3 deposition assay” section using a rabbit monoclonal anti-His antibody (Santa Cruz, cat. no. sc-803, 1:1000) as the primary antibody and goat anti-rabbit IgG conjugated with Cy3 (Jackson Immuno Research Laboratories Inc., cat. no. 111-165-144, 1:100).

### Mutational analysis of Set2 in yeast

The *S. cerevisiae* strains used in this study are shown in table S4. The *set2* deletion strain was prepared by replacing the endogenous *SET2* gene with the kanamycin resistance gene (*kanMX6*) in the BY4741 strain (genotype: *MATa his3*Δ*1 leu2*Δ*0 met15*Δ*0 ura3*Δ*0*). To construct *SET2* WT and mutants in the *SET2* deletion background, the *SET2* deletion strain (*set2*∆*::kanMX6*) was transformed with DNA fragments encoding *SET2*-3× FLAG [*SET2* (*WT*), *set2* (*5A*), *set2* (Δ*SRI*), or *set2* (*5A-*Δ*SRI*)] and *URA3* genes. The DNA fragments were synthesized by Twist Bioscience. The strains were selected on yeast synthetic complete medium without uracil [SC-uracil: Difco yeast nitrogen base without amino acids (6.7 g liter^−1^; BD Biosciences, 291940), yeast Synthetic Drop-out medium supplements (1.92 g liter^−1^; Merck, Y1501-20G), 2% glucose, and 2% Difco Bacto Agar].

The yeast cells were grown at 30°C in YPD medium (YPD: 1% yeast extract, 2% peptone, and 2% glucose) overnight, and then the culture was diluted twofold by adding fresh YPD medium 2 hours before harvest. After centrifugation, the supernatant was removed. The resulting pellet was suspended in 250 μl of ice-cold water, and 37.5 μl of lysis buffer [2 M NaOH and 7.5% 2-mercaptoethanol] was added. After incubation for 10 min, 37.5 μl of 50% (v/v) trichloroacetic acid was added. The samples were centrifuged, and the supernatants were removed. The resulting pellets were dissolved in SDS buffer and heated at 65°C for 5 min.

The extracted samples were fractionated by SDS-PAGE (Nacalai Tesque, Extra PAGE One Precast Gel 10 to 20%, cat. no. 13068-24). PageRuler Plus (Thermo Fisher Scientific, cat. no. 26619) protein markers were used. After electrophoresis, the proteins in the gel were transferred to a membrane (Cytiva, Amersham Protran Premium NC 0.2 μm, cat. no. 10600009). The membrane was blocked with Blocking One-P (Nacalai Tesque, cat. no. 05999-84) or Blocking One (Nacalai Tesque, cat. no. 03953-66). After washing with TBS-T, the membrane was incubated with a mouse monoclonal anti-H3K36me3 antibody (Active Motif, cat. no. 61022, 1:1000) overnight at 4°C, a rat anti-Tubulin alpha antibody with HRP (horseradish peroxidase) (Bio-Rad, cat. no. MCA77P, 1:5000) overnight at 4°C, or a mouse anti-FLAG antibody (Sigma-Aldrich, cat. no. F3165, 1:5000) for 1 hour at room temperature. These antibodies were diluted with Can Get Signal Solution 1 (TOYOBO, cat. no. NKB-201). For H3K36me3 detection, after washing with TBS-T, the membrane was incubated with goat anti-mouse IgG conjugated with Cy3 (Jackson Immuno Research Laboratories Inc., cat. no. 115-165-146, 1:100) for 1 hour at room temperature. The fluorescence signals of the membrane were detected by an Amersham Typhoon imager. For tubulin detection, the chemical luminescence signal was detected using ECL Prime Reagents (Cytiva) by an Amersham Imager 680. For FLAG detection, after washing the membrane with TBS-T, the membrane was incubated with goat anti-IgG (H + L chain) Mouse pAb-HRP (MBL no. 330, 1:5000) for 1 hour at room temperature. The chemical luminescence signal was detected using ECL Prime Reagents (Cytiva) by an Amersham Imager 680.

### Preparation of EC-nucleosome complexes for cryo-EM analysis

For the EC115-Set2 complex containing H2BK120Cub, the transcription reaction was conducted by mixing the Temp115 nucleosome containing H3.3 (K36M) and H2B (K120Cub), RNAPII, TFIIS, Spt4/5, Elf1, Paf1C, Spt6, Spn1, FACT, P-TEFb, CK2α, Set2, and a DY647 fluorescently labeled RNA primer in 720 μl of reaction solution containing *S*-adenosyl-l-homocysteine, UTP, GTP, CTP, and 3′-dATP and incubating at 30°C for 75 min. The resulting reaction mixture contains the 0.1 μM template nucleosome, 0.1 μM RNAPII, 0.1 μM TFIIS, 0.4 μM Spt4/5, 1.0 μM Elf1, 0.2 μM RNA, 0.27 μM CK2α, 0.1 μM P-TEFb, 0.4 μM Spt6, 0.5 μM Spn1, 0.4 μM Paf1C, 0.5 μM FACT, and 1.0 μM Set2 in 31 mM Hepes-KOH (pH 7.5) buffer containing 68 mM KOAc, 0.4 μM Zn(OAc)_2_, 0.04 mM TCEP, 2.5% glycerol, 15 mM NaCl, 0.2 mM DTT, 5 mM MgCl_2_, 0.02 mM EDTA, 0.1 mM *S*-adenosyl-l-homocysteine, 0.4 mM UTP, 0.4 mM GTP, 0.4 mM CTP, and 0.4 mM 3′-dATP. The resulting mixture was fractionated by the GraFix method (*55*). The sucrose gradient solution was prepared by a Gradient Master instrument (SKE) using sucrose-low buffer [20 mM Hepes-KOH (pH 7.5), 50 mM KOAc, 0.2 μM Zn(OAc)_2_, 0.1 mM TCEP, and 10% sucrose] and sucrose-high buffer [20 mM Hepes-KOH (pH 7.5), 50 mM KOAc, 0.2 μM Zn(OAc)_2_, 0.1 mM TCEP, 25% sucrose, and 0.1% glutaraldehyde].

For the EC115-Set2 complex without H2BK120Cub, the sample was prepared as described above with the Temp115 nucleosome containing H3.3 (K36M) and H2B (WT). For the EC130-Set2 complex, the sample was prepared as described above with the Temp130 nucleosome containing H3.3 (K36M) and H2B (WT).

The reaction solution was applied on top of the gradient solution and centrifuged at 27,000 rpm at 4°C for 16 hours using an SW41 rotor (Beckman Coulter). The fractions containing the EC-nucleosome complex were collected and dialyzed against 20 mM Hepes-KOH (pH 7.5) buffer containing 20 mM KOAc, 0.2 μM Zn(OAc)_2_, and 0.1 mM TCEP. After dialysis, the sample was concentrated by an Amicon Ultra 100K centrifugal concentrator (Millipore). For vitrification, the sample was supplemented with 0.0025% Tween 20 and applied to Quantifoil grids (copper, R1.2/1.3, 200 mesh; Quantifoil Micro Tools). The grids were glow discharged before the sample application for 2 min by a PIB-10 ION Bombarder (Vacuum Device Inc.). The grids were blotted with grade 595 filter paper (Ted Pella) and then plunge-frozen in liquid ethane using a Vitrobot Mark IV (Thermo Fisher Scientific) at 4°C and 100% humidity.

### Cryo-EM data collection and image processing

All cryo-EM data were collected with a Krios G4 transmission electron microscope (Thermo Fisher Scientific) equipped with a BioQuantum energy filter and a K3 camera (Gatan). The data collection was performed using EPU (Thermo Fisher Scientific), with a calibrated pixel size of 0.83 Å per pixel. Subsequent image processing was accomplished with Relion 3.1 (*56*), unless otherwise specified.

The initial stages of image processing were done similarly for all datasets, as described previously (*20*). Briefly, the initial particle picking was performed with Relion blob picker. After preliminary two-dimensional (2D) and 3D classifications, Topaz networks were trained with particles from several good classes, and then another round of particle picking was performed with the newly trained networks. After roughly removing bad particles with 2D and 3D classifications, all particle sets were merged and then further purified by 2D and 3D classifications. The particles were then reextracted with lower binning, and Bayesian polishing and contrast transfer function refinements were performed to improve the data quality. Through these steps, high-quality particle stacks centered at RNAPII were first obtained.

To analyze the molecular structures upstream of the Temp115 dataset, the particles were first subjected to 3D classifications using a mask around the EC, and then the particles with a strong density for Spt6 were selected (fig. S4D). These particles were subjected to multiple local 3D classifications mainly focused on the upstream region of the EC, which led to the structural solutions of the two EC115-Set2 complexes (fig. S5) and the EC115^hex^-Set2-FACT complex (fig. S7). In addition to the overall reconstruction, upstream reconstruction and nucleosome reconstruction were prepared using Blush regularization implemented in Relion5 (*57*).

To obtain a higher-resolution map of the Spt6-Set2 interface, particles with Spt6 and Set2 were pooled and subjected to multiple 3D classifications around Spt6 using Blush regularization (fig. S6A). This resulted in the 3.06-Å reconstruction of Spt6 bound with the Set2 YKIPK peptide and the 3.77-Å reconstruction of Spt6 bound with Set2 (AID). Also, to better visualize Set2 (CD) bound to the nucleosome, upstream particles containing a suitable nucleosome and Set2 (CD) were collected and then subjected to multiple 3D classifications around Set2 (CD) using Blush regularization (fig. S6B). This resulted in the 4.11-Å reconstruction of nucleosome-Set2 (CD).

The image processing of the Temp115 without H2BK120Cub [Temp115(noUb)] dataset was done in essentially the same way as the Temp115 dataset. Two distinct maps for the EC115-Set2 complex without H2BK120Cub were obtained, which were virtually indistinguishable from those derived from the Temp115 with H2BK120Cub dataset.

For the Temp130 dataset, the particles with a strong density for Spt6 were first selected, as was done for the Temp115 datasets. Then, with 3D classifications focused on the upstream region, two distinct classes (classes A and B) for the upstream nucleosome were obtained (fig. S15). At the same time, independently from the upstream classifications, another cycle of 3D classification with a mask around the EC was performed, which led to cryo-EM maps with a stronger density for Paf1C. The particles that belonged to both strong-Paf1 classes and respective upstream nucleosome classes were selected. For nucleosome class A, another round of 3D classification around the nucleosome was performed to further improve the quality. Last, EC reconstructions and nucleosome reconstruction with Blush were prepared, in addition to the overall reconstruction.

### Model building

Because of the structural flexibility between the EC region and the nucleosome region, each region was first modeled independently using the respective local reconstruction with higher quality. Then, the partial models were assembled into the upstream reconstructions (for Temp115 structures) or the overall reconstructions (for Temp130 structures). The model assembly was performed manually using WinCoot (*58*), ChimeraX (*59*), and ISOLDE (*60*), and then the final model was refined using Phenix (*61*).

To build the EC region including Set2 (AID) of the Temp115 dataset, the previous EC structure [Protein Data Bank (PDB): 7XN7] was used as the starting model (*20*). As the map qualities of some regions (especially the RNAPII core) in the current reconstruction were better than the previous ones, some of these regions were edited manually and then refined with Phenix, which led to the EC model with better statistics. For all complexes (Set2^A^, Set2^B^, and the FACT-hexamer complex), the cryo-EM density for the DNA-RNA hybrid in the RNAPII active site was consistent with that of EC115 (fig. S9C). Spt6 and its adjacent regions were manually edited and refined with Phenix using the Spt6 reconstruction. The Set2 peptide region bound to Spt6 was modeled manually and refined with Phenix. For the Set2 (AID) region, the model was first built with AlphaFold2 (*62*), which was fit into the Spt6-Set2 (AID) reconstruction, and then further refined with Phenix.

To build the nucleosome bound with Set2 (CD) of the EC115-Set2^(A/B)^ structures, the nucleosomal region of the EC115 structure (PDB: 7XSZ) from the previous work was used as the starting model (*20*). The initial model for Set2 (CD) was created with AlphaFold2 and was fit into the nucleosome-Set2 (CD) reconstruction. The model was edited manually with WinCoot, ChimeraX, and ISOLDE and then refined using Phenix. After this, the Set2 (CD)-nucleosome models were slightly adjusted for Set2^A^ and Set2^B^ using their respective upstream and nucleosomal reconstructions. The nucleosome region of the upstream FACT-hexamer was based on the previous structure (PDB: 7XTI), which was manually adjusted and refined with Phenix.

The EC region of the Temp130 structures was very similar to that of the Temp115 structures, except for the nucleotides and the slight reorientation of Set2 (AID). The cryo-EM density for the DNA-RNA hybrid showed that there was a 1-bp shift in the nucleotide register between 130^A^ and 130^B^ (fig. S16D), and models were adjusted accordingly. In the 130^B^ structure, the density for the Leo1 C-terminal helix was missing, possibly due to the strongly bent upstream DNA, and the corresponding Leo1 residues were deleted. Nucleosome regions were modeled starting from the nucleosome-Set2 (CD) model of the 115-Set2^B^ structure, which was manually edited and refined using nucleosome reconstruction.
