# Supplementary material for: Structural basis of transcription-coupled H3K36 trimethylation by Set2 in coordination with FACT
Source: Sci Adv. 2026 Jan 28;12(5):eaed1952. doi: 10.1126/sciadv.aed1952 (PMC12851032; doi:10.1126/sciadv.aed1952)
Supplement: Supplementary file 1 — Figs. S1 to S19 Tables S1 to S6 Legend for movie S1 [file sciadv.aed1952_sm.pdf]

Science Advances
AAAS

Supplementary Materials for

Structural basis of transcription-coupled H3K36 trimethylation by Set2 in coordination with FACT

Tomoya Kujirai *et al.*

Corresponding author: Hitoshi Kurumizaka, kurumizaka@iqb.u-tokyo.ac.jp;
Shun-ichi Sekine, shunichi.sekine@riken.jp

Sci. Adv. **12**, eaed1952 (2026)
DOI: 10.1126/sciadv.aed1952

The PDF file includes:

Figs. S1 to S19
Tables S1 to S6
Legend for movie S1

Other Supplementary Material for this manuscript includes the following:

Movie S1

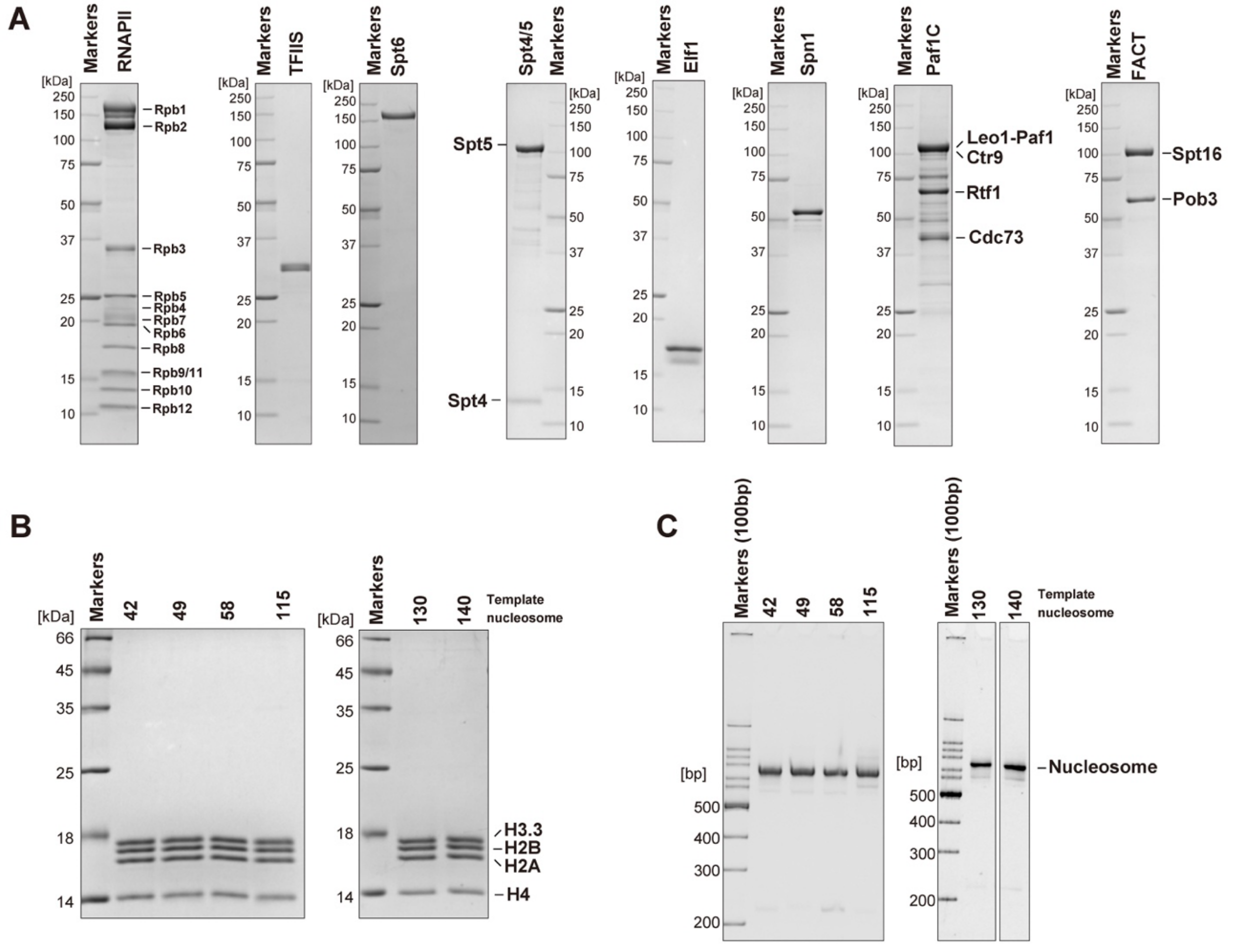

Fig. S1. Protein preparation. (**A**) SDS-PAGE gels of RNAPII, transcription elongation factors, and FACT. (**B**) SDS-PAGE gel of the template nucleosomes. (**C**) Native-PAGE gel of the template nucleosomes. The SDS-PAGE and native-PAGE gels were stained with Coomassie Brilliant Blue and ethidium bromide, respectively.

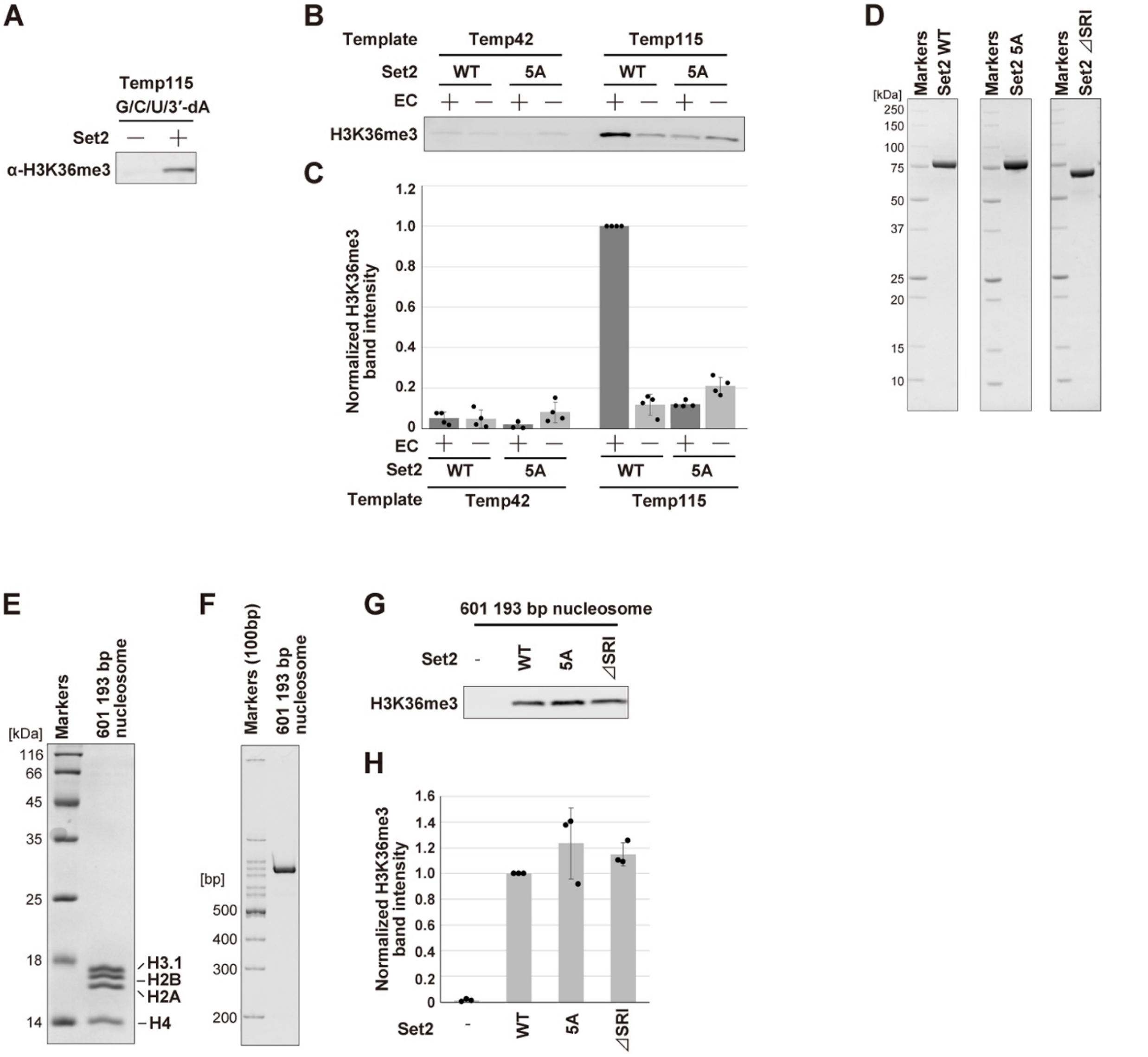

Fig. S2. H3K36me3 deposition by Set2 under various conditions. (**A**) Transcription-coupled H3K36me3 deposition in the presence or absence of Set2. This experiment was performed in triplicate. (**B**) Western blot of H3K36me3 deposition in the presence or absence of EC components (RNAPII, TFIIS, Spt4/5, Elf1, Paf1C, Spt6, Spn1, and P-TEFb**)** in Temp42 or Temp115. (**C**) Quantification of panel (B). The mean values (bars) of relative signals compared to the Temp115/Set2 (WT) are shown with S.D. (error bars, n=4). (**D**) SDS-PAGE gel of the Set2 mutants. (**E**) SDS-PAGE gel of the nucleosome containing the Widom601 193 base-pair DNA. (**F**) Native-PAGE gel of the nucleosome containing the Widom601 193 base-pair DNA. The SDS-PAGE and native-PAGE gels were stained with Coomassie Brilliant Blue and ethidium bromide, respectively. (**G**) Western blot of H3K36me3 deposition without transcription by Set2 mutants on the nucleosome containing the Widom601 193 base-pair DNA. (**H**) Quantification of panel (G). The mean values (bars) of the relative signals compared to the Set2 (WT) are shown with S.D. (error bars, n=3).

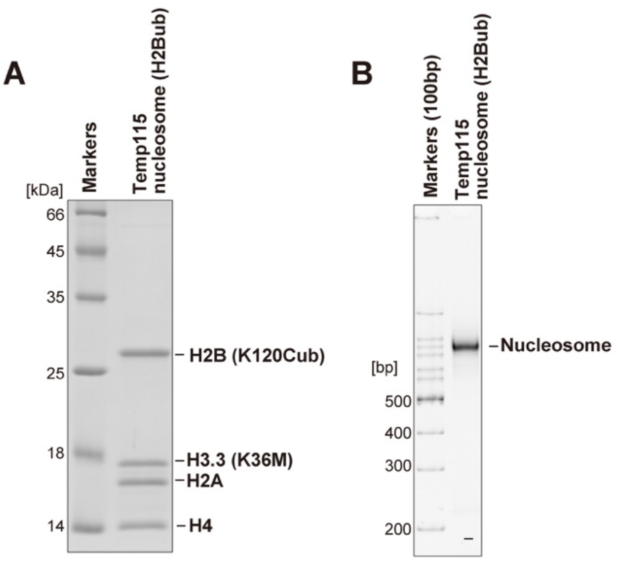

Fig. S3. Preparation of EC115-Set2 for the cryo-EM analysis. (**A**) SDS-PAGE of the nucleosome containing H2A, H2B (K120Cub), H3.3 (K36M), and H4. (**B**) Native-PAGE of the nucleosome. The SDS-PAGE and native-PAGE gels were stained with Coomassie Brilliant Blue and ethidium bromide, respectively. (**C**) Urea-PAGE of the elongated RNA in the EC115-Set2 complex. The fluorescence signal of DY647 dye conjugated to the RNA was detected.

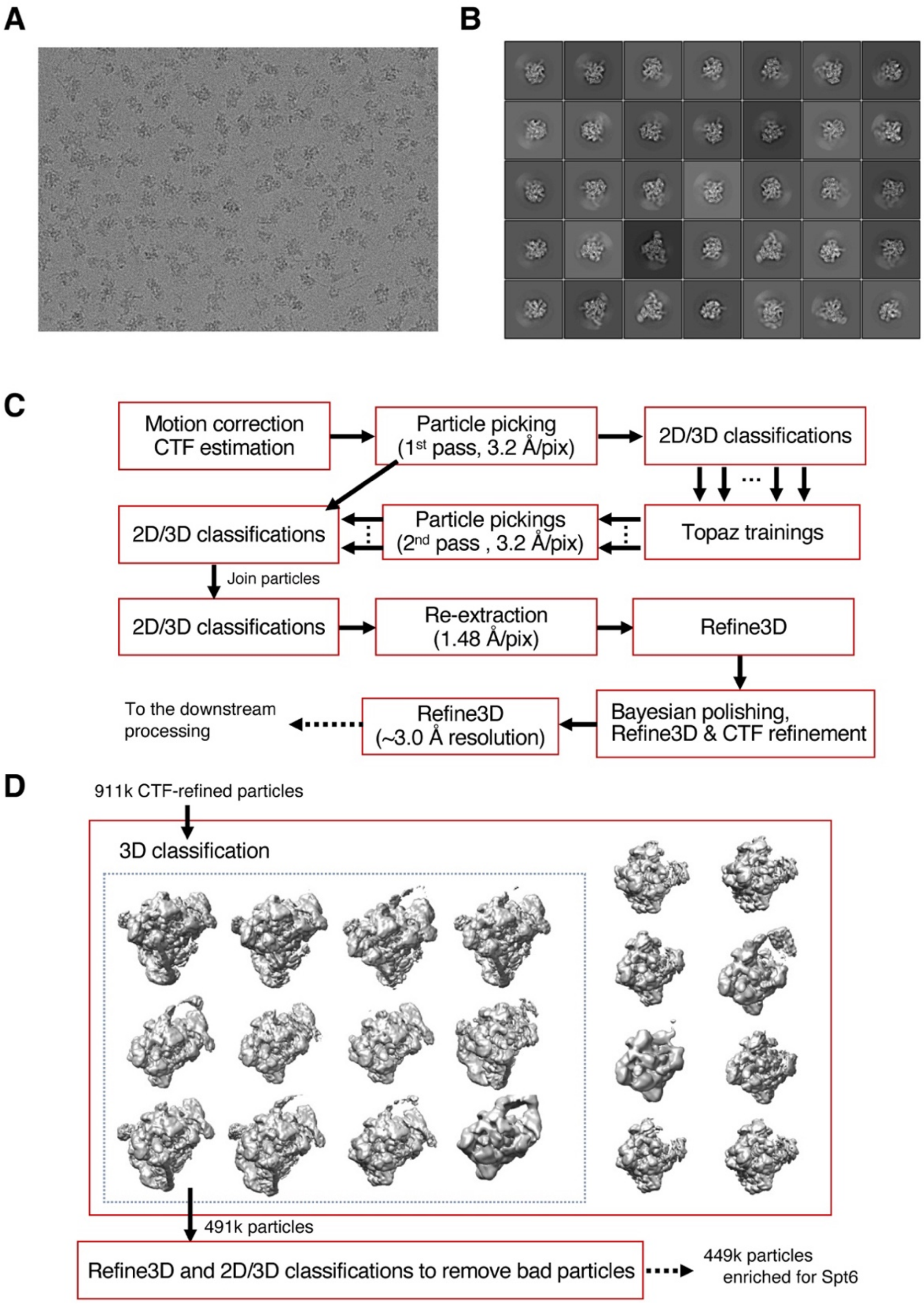

Fig. S4. Cryo-EM data collection and initial image processing of EC115-Set2. (**A**) Representative cryo-EM micrograph. (**B**) Representative 2D class averages from reference-free 2D classification after the Spt6-containing particles were enriched. (**C**) Representative flowchart describing the initial stage of image processing. (**D**) Representative 3D classification performed to enrich Spt6-containing particles (from one of the processing batches).

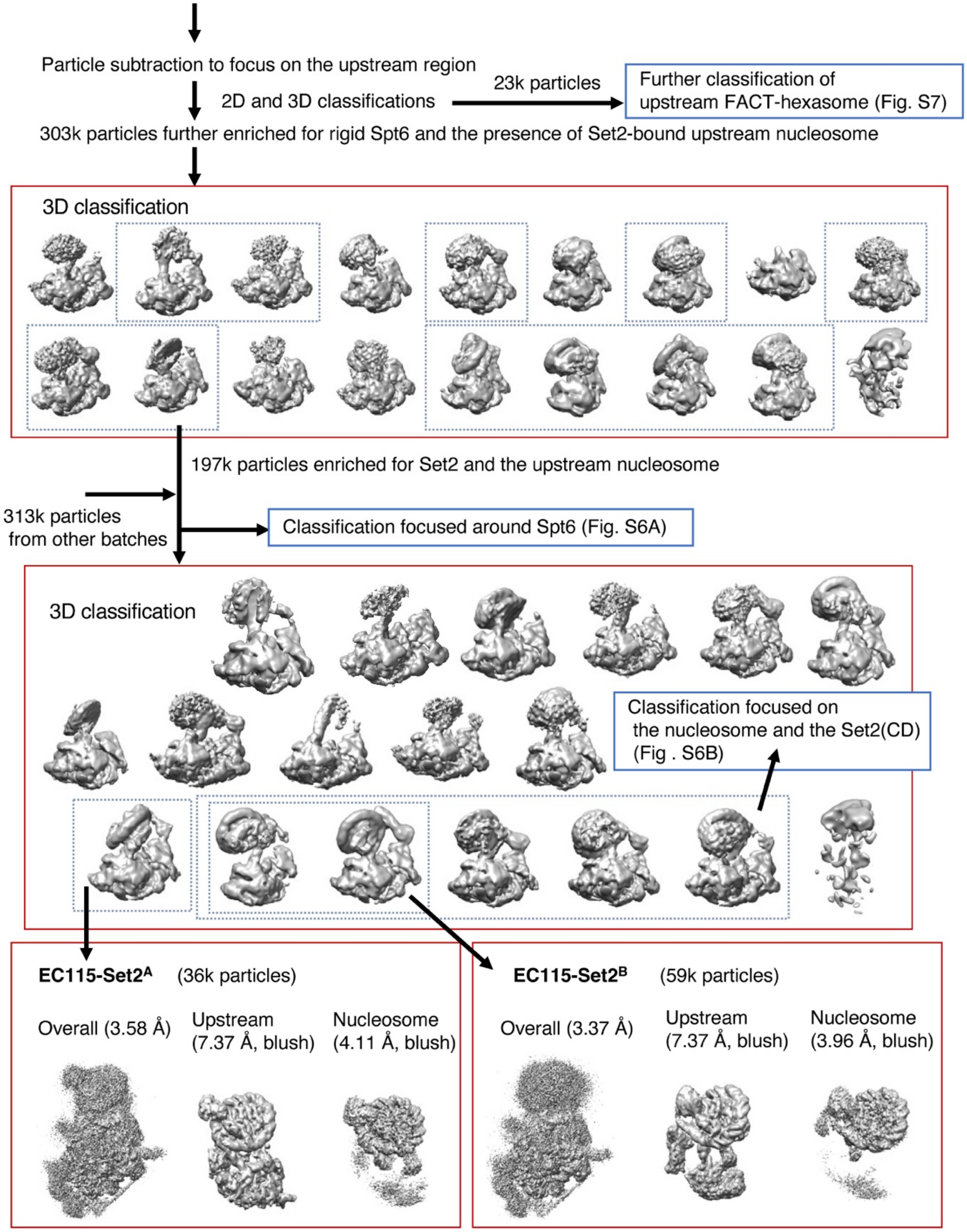

Fig. S5. Cryo-EM data analysis leading to the EC115-Set2 structures.

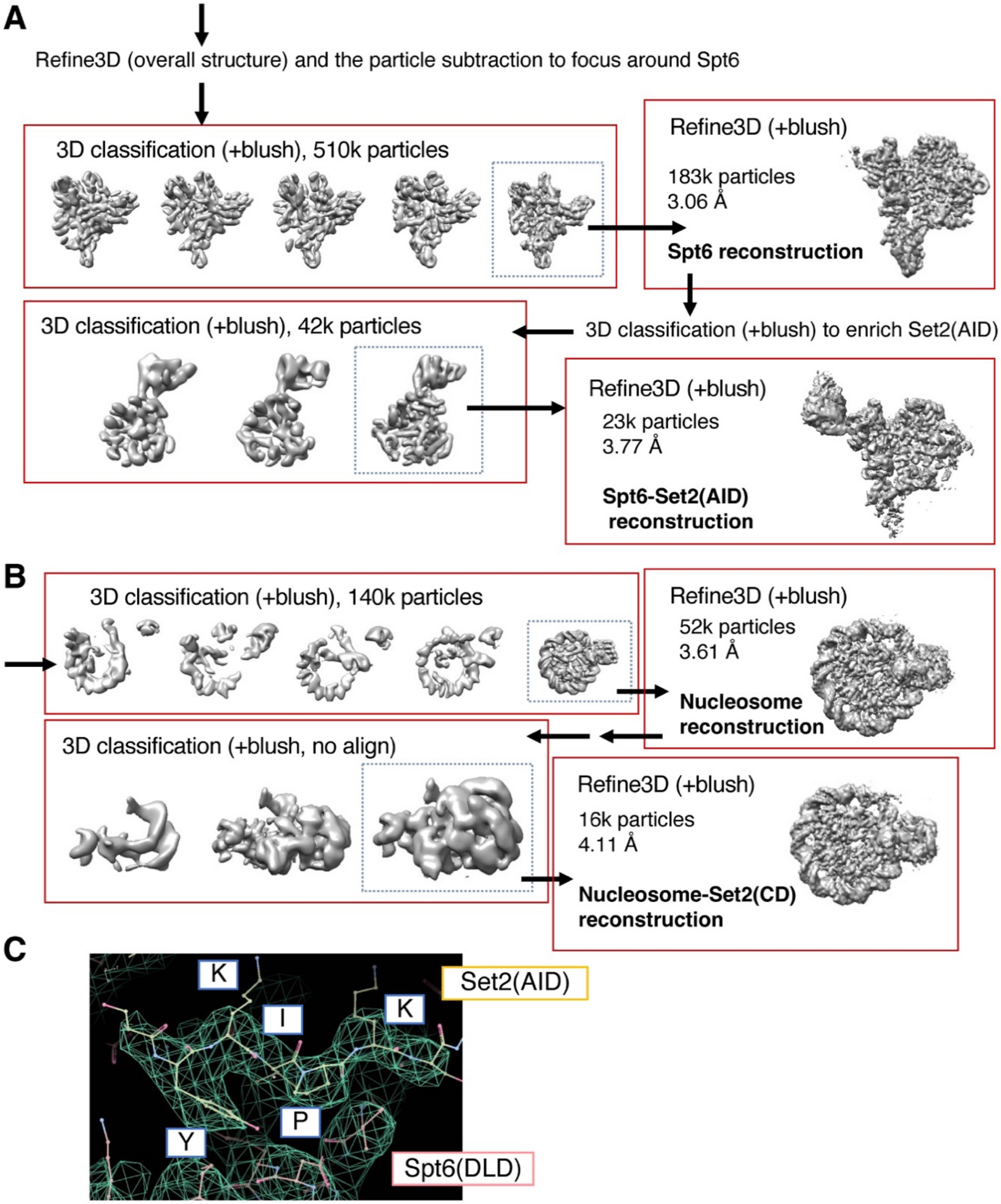

Fig. S6. Cryo-EM data analysis leading to the local reconstructions around Set2 and Spt6 of EC115-Set2. (**A**) Classifications leading to the Spt6-Set2 (AID) reconstruction. (**B**) Classifications leading to the Nucleosome-Set2 (CD) reconstruction. (**C**) Cryo-EM map around the Set2 YKIPK motif from the Spt6 reconstruction. The map was sharpened and resampled in WinCoot.

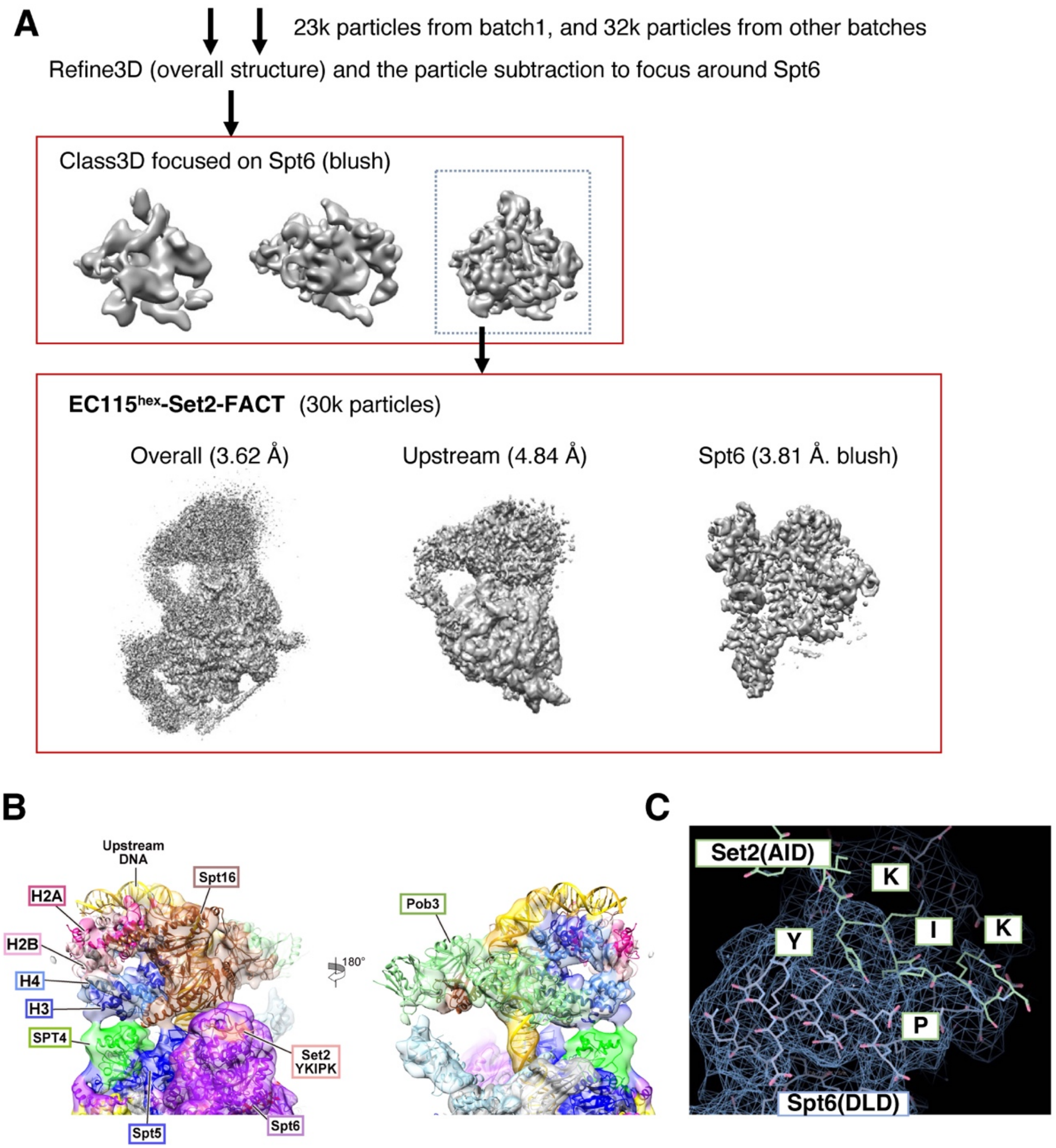

Fig. S7. Structure of EC115^{hex}-Set2-FACT. (**A**) Classifications leading to the EC115^{hex}-Set2-FACT structure. (**B**) Cryo-EM density of the upstream region of EC115^{hex}-Set2-FACT fitted with the atomic model. The EM map was visualized with a 9 Å lowpass filter. (**C**) Cryo-EM map around the Set2 YKIPK motif. The map was sharpened and resampled in WinCoot.

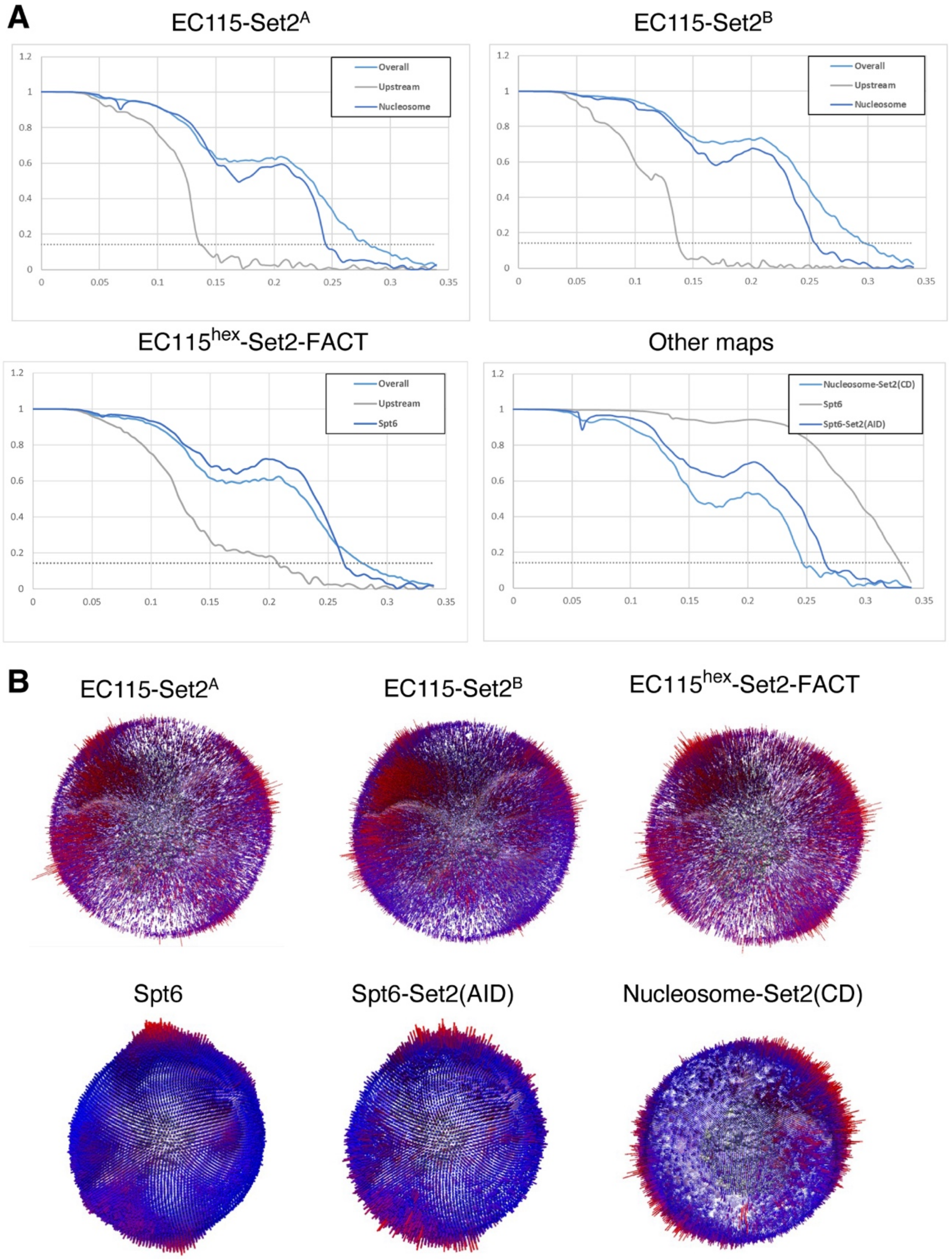

Fig. S8. Cryo-EM statistics of EC115-Set2 reconstructions. (**A**) Gold-standard Fourier shell correlation (FSC) curves of the EC115-Set2 complexes, and related local reconstructions. FSCs were calculated by Relion Refine3D, and dashed lines represent the FSC threshold of 0.143. (**B**) Orientation distributions of the cryo-EM reconstructions.

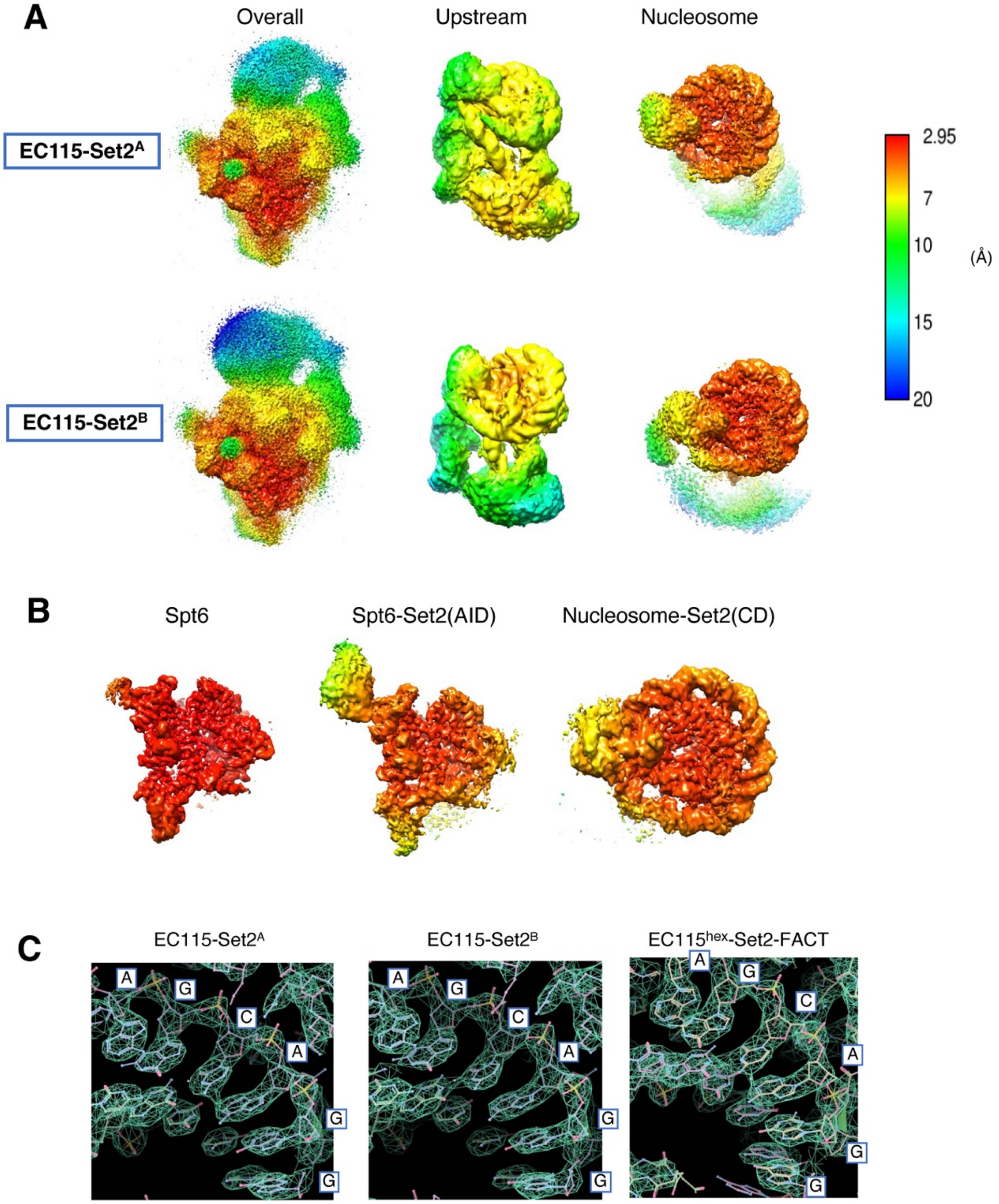

Fig. S9. Details of the EC115-Set2 structures. (**A**) Local resolutions for EC115 -Set2 complexes. (**B**) Local resolutions for the reconstructions around Set2, Spt6, and nucleosome. Local resolutions were calculated by Relion, with 30 Å sampling. (**C**) Cryo-EM densities of the EC115-Set2. Cryo-EM maps around the EC active site from the EC115-Set2 complexes (overall reconstruction). The map is sharpened and resampled in WinCoot.

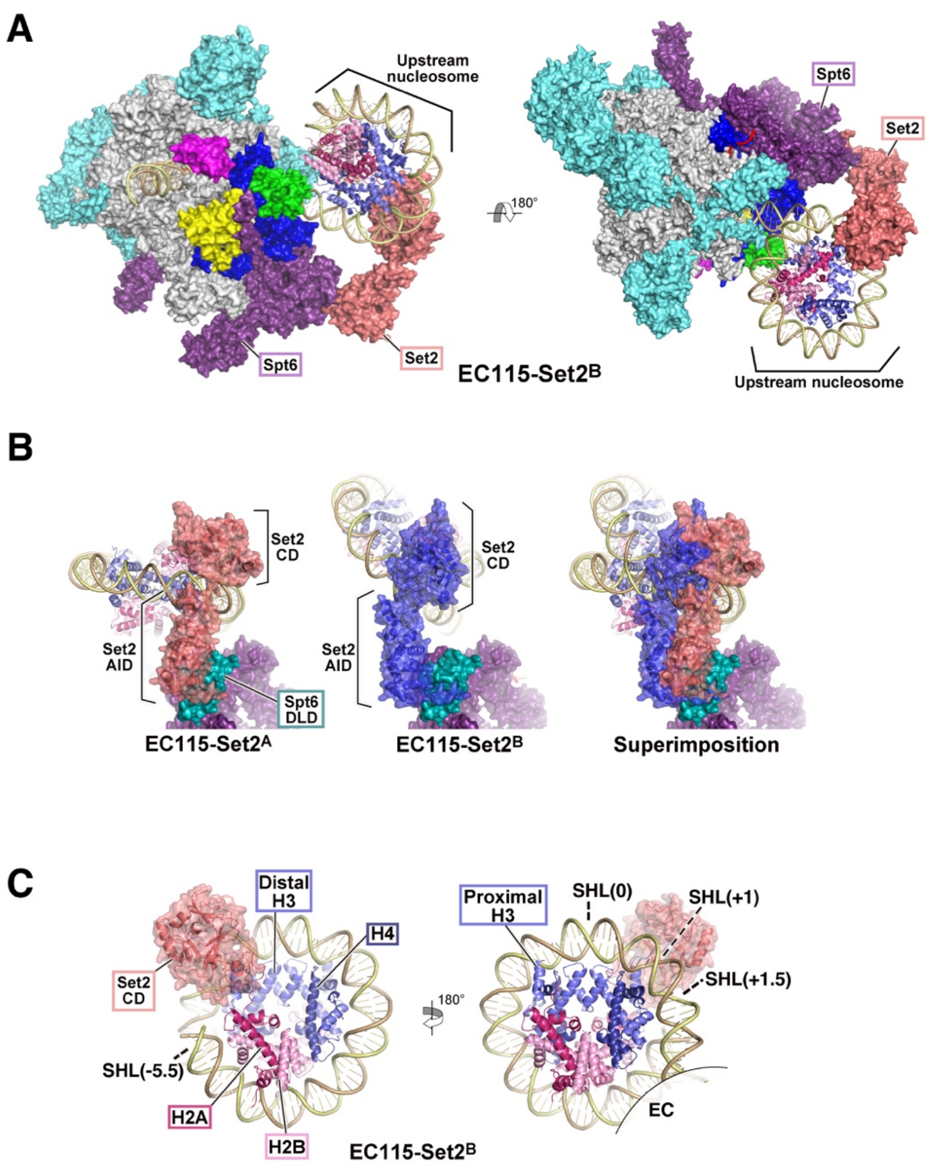

Fig. S10. EC115-Set2^{B} structure. (**A**) Overall structure of the EC115-Set2^{B}. The EC and Set2 structures are shown in surface models. The nucleosome structure is shown in a ribbon model. (**B**) Comparison of the Set2 structures in the EC115-Set2^{A} and EC115-Set2^{B} complexes. (**C**) Structure of the nucleosome with the Set2 CD contained in the EC115-Set2^{B}. The CD is shown in a ribbon model with a transparent surface model.

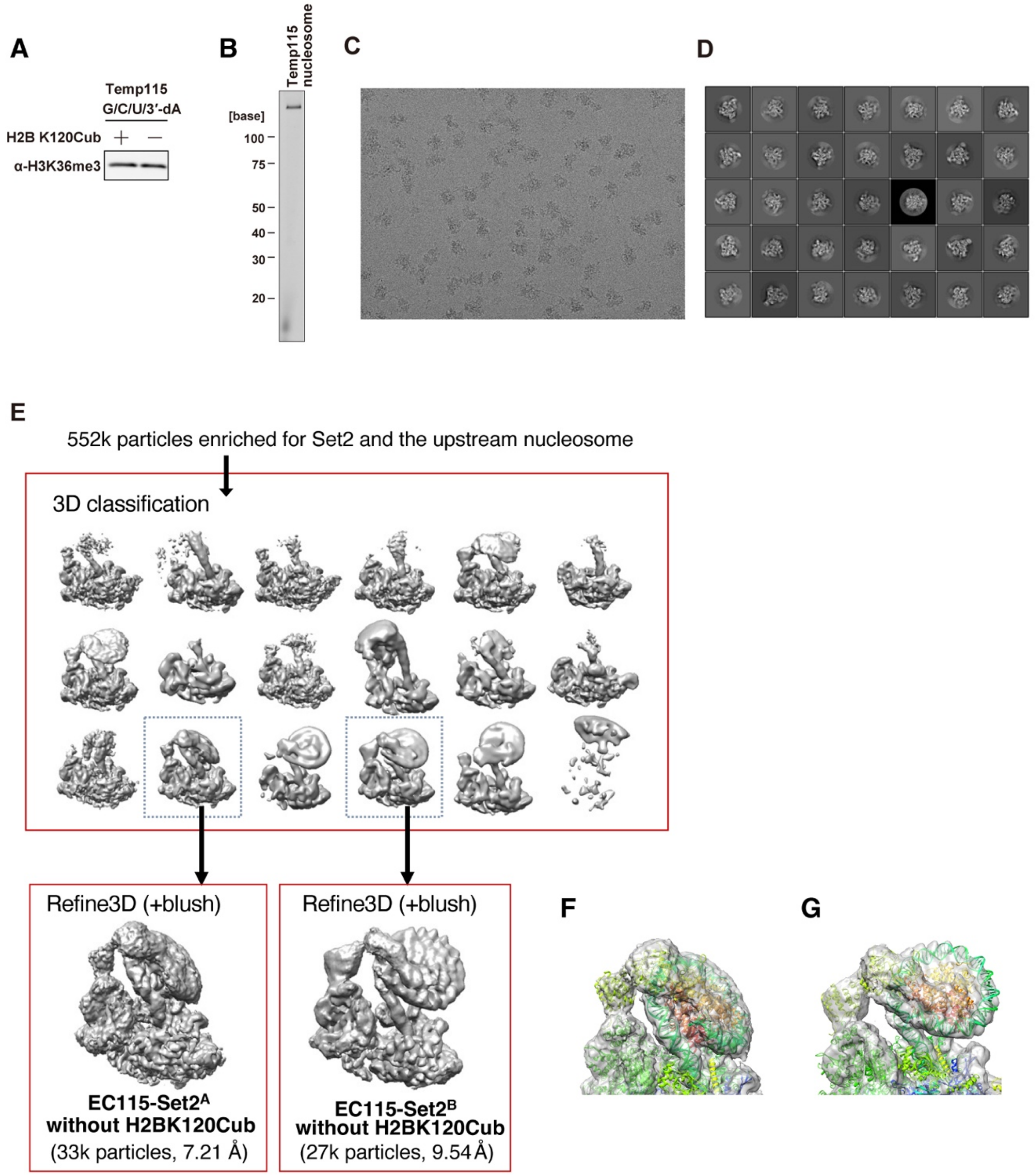

Fig. S11. Influence of H2B ubiquitination on EC115-Set2. (**A**) Western blot of H3K36me3 deposition by Set2, in the presence or absence of H2BK120Cub. This experiment was performed in duplicate. (**B**) Urea-PAGE of the elongated RNA in EC115-Set2 without H2BK120Cub, which was used for cryo-EM analysis. The fluorescence signal of DY647 dye conjugated to the RNA was detected. (**C**) Representative cryo-EM micrograph. (**D**) Representative 2D class averages from reference-free 2D classification after the Spt6-containing particles were enriched. (**E**) Representative flowchart describing the initial stage of image processing. (**F**, **G**) The EM densities of EC-Set2^{A} (F) and EC115-Set2^{B} (G) without H2BK120Cub are fitted with the atomic models of EC115-Set2 with H2BK120Cub.

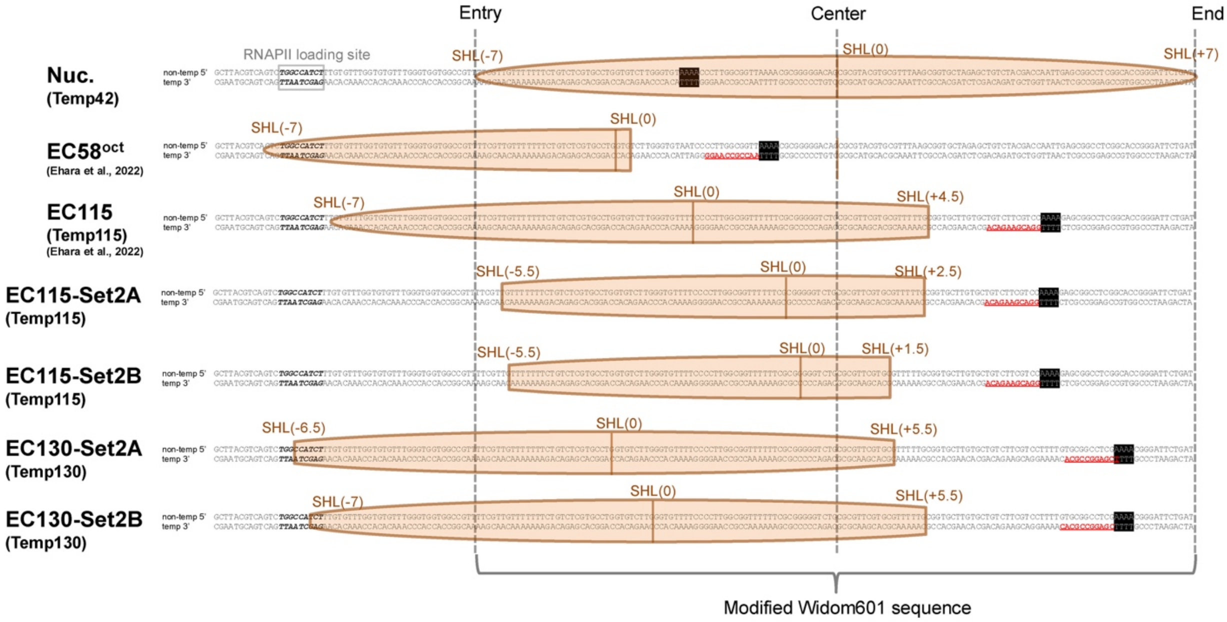

Fig. S12. Nucleosome positions on the template DNA. The positions of the EC and the nucleosome in the EC structures. The approximate nucleosome positions and nucleosome centers (SHL(0)) are indicated by orange circles and vertical lines, respectively. The DNA-RNA hybrid region within the RNAPII active site is colored red and underlined. The positions of the T stretches to stall the EC are highlighted by a black background. The RNAPII loading site is indicated in italics.

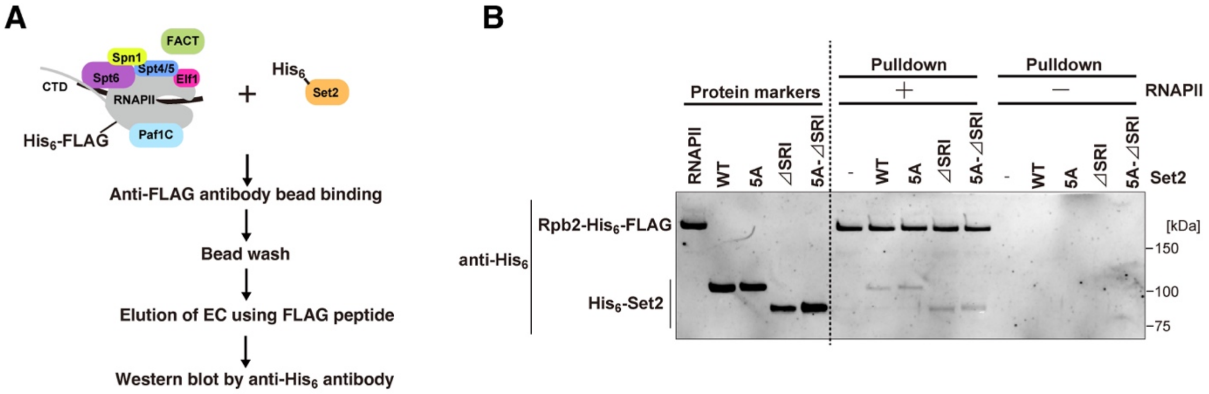

Fig. S13. Set2 association to EC. (**A**) Scheme of the Set2 pulldown assay. The reconstituted EC containing His_6-FLAG-tagged Rpb2, an RNAPII subunit, was mixed with anti-FLAG antibody beads in the presence of His_6-tagged Set2 protein. After the EC-bound beads were washed, the EC was eluted using FLAG peptide and the EC-associated Set2 was detected by western blotting. (**B**) Western blot of the Set2 pulldown assay. The EC-associated His_6-Set2 mutants were detected by an anti-His_6 antibody. This experiment was performed in duplicate.

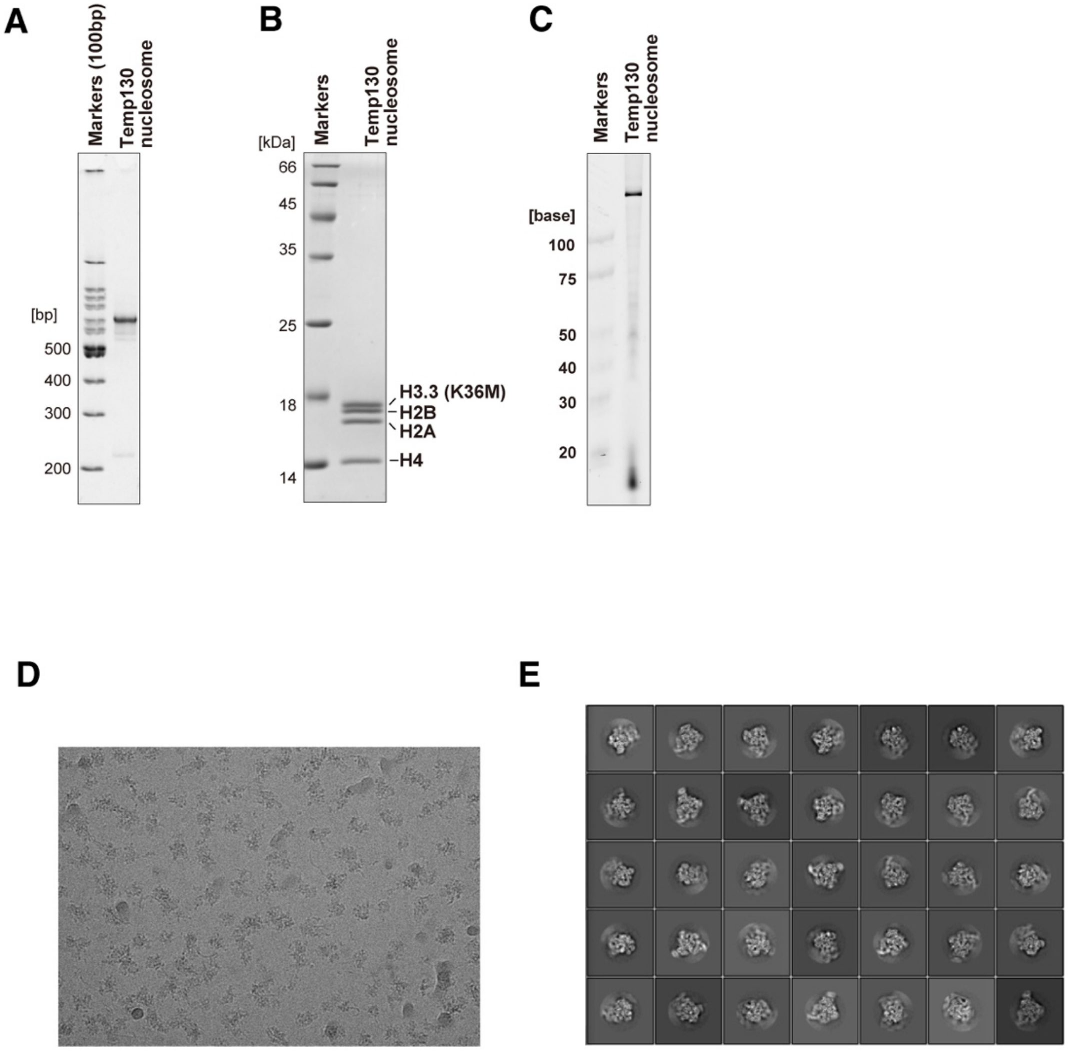

Fig. S14. Sample preparation and cryo-EM data collection of EC130-Set2. (**A**) Native-PAGE gel of the template nucleosomes. (**B**) SDS-PAGE gel of the template nucleosomes. (**C**) Urea-PAGE of the elongated RNA in the EC130-Set2 complex. The fluorescence signal of DY647 dye conjugated to the RNA was detected. (**D**) Representative cryo-EM micrograph. (**E**) Representative 2D class averages from reference-free 2D classification after the Spt6-containing particles were enriched.

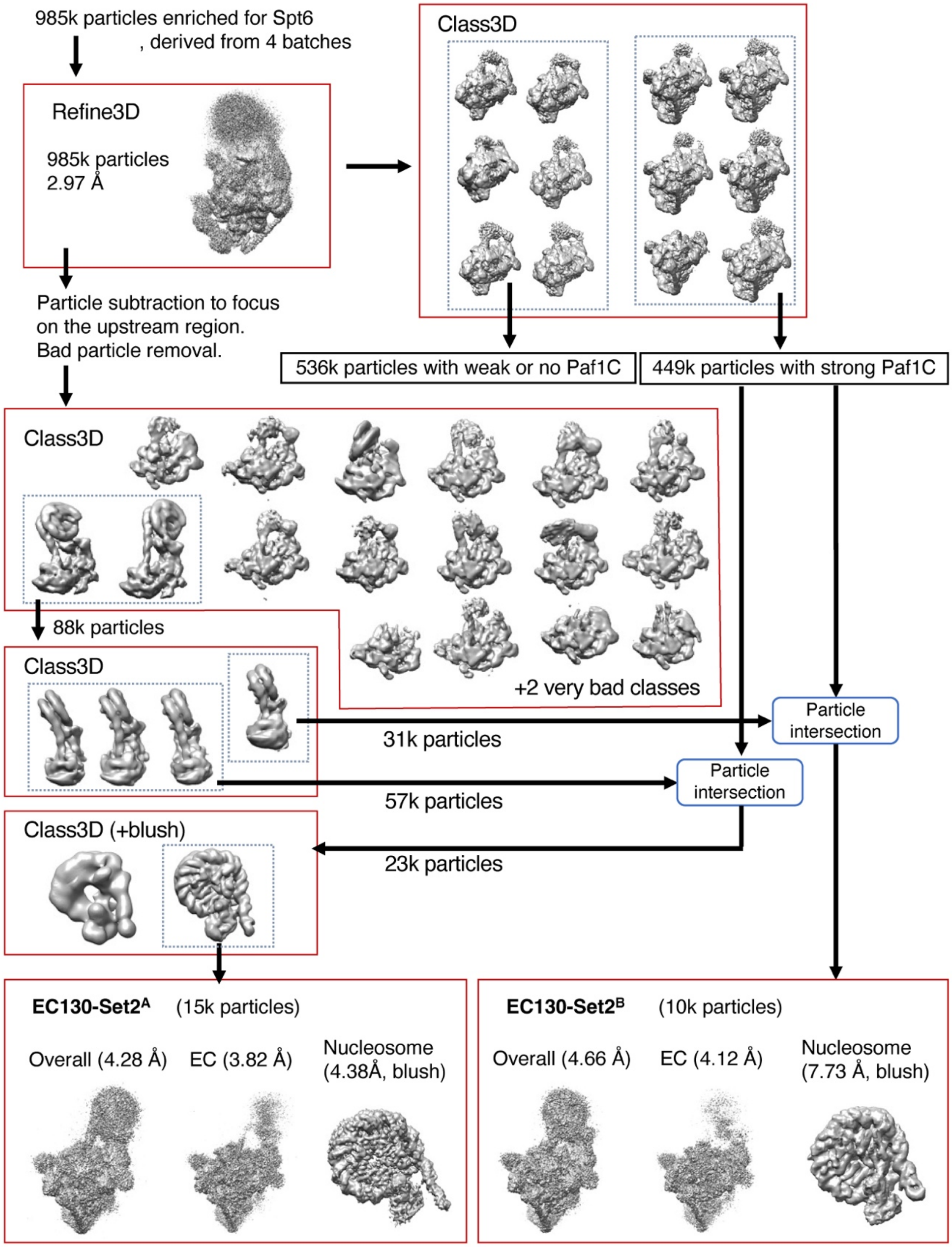

Fig. S15. Cryo-EM data analysis leading to the EC130-Set2 structures.

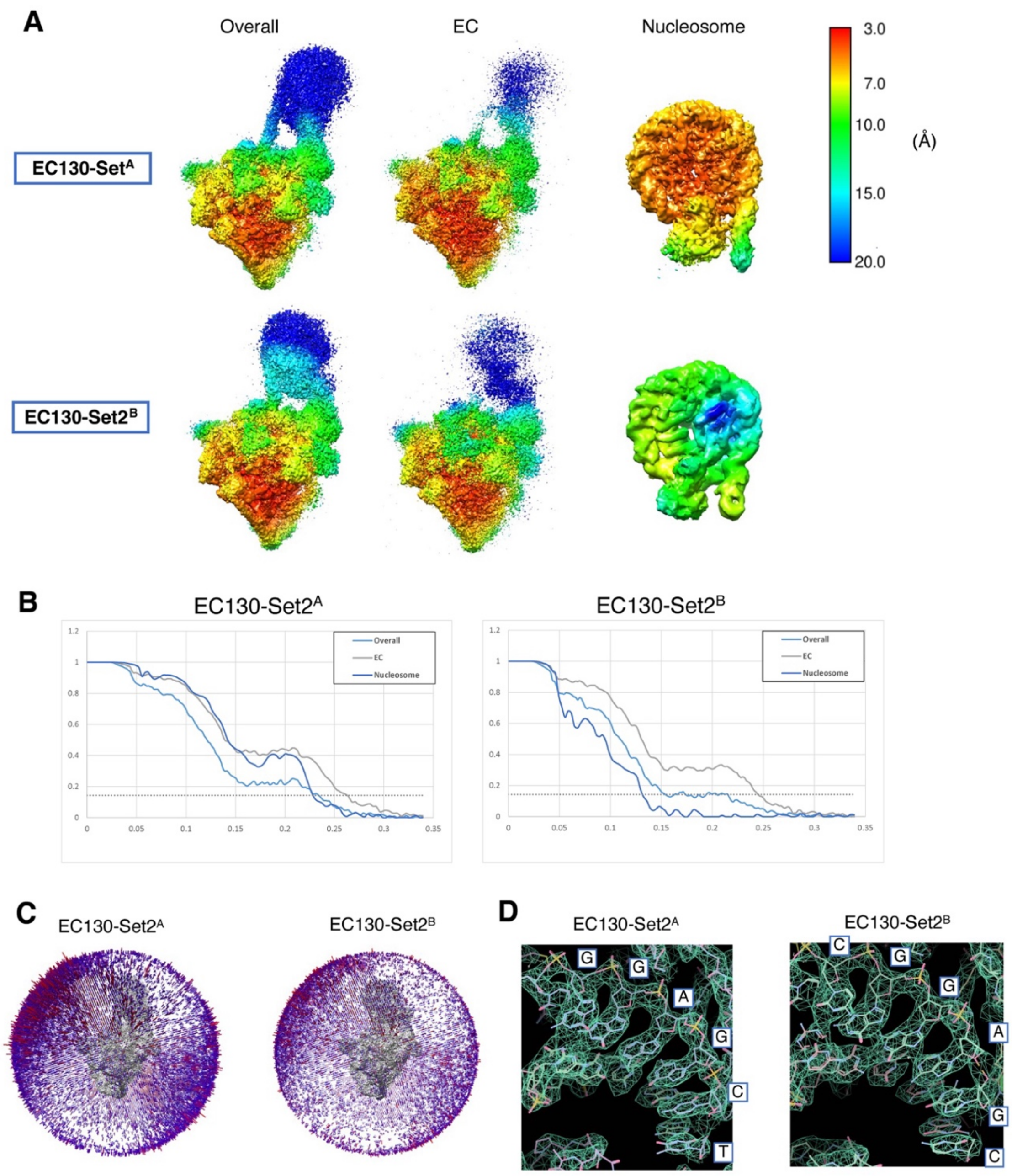

Fig. S16. Details of the EC130-Set2 structures. (**A**) Local resolutions for EC115-Set2 complexes. Overall (left), EC (middle), and nucleosome (right) are shown. (**B**) Gold-standard Fourier shell correlation (FSC) curves of the EC130-Set2 complexes. FSCs were calculated by Relion Refine3D, and dashed lines represent the FSC threshold of 0.143. (**C**) Orientation distributions of the cryo-EM reconstructions of EC130-Set2^{A} (left) and EC130-Set2^{B} (right). (**D**) Cryo-EM maps around the EC active site from the EC130-Set2 complexes (overall reconstruction). The map was sharpened and resampled in WinCoot.

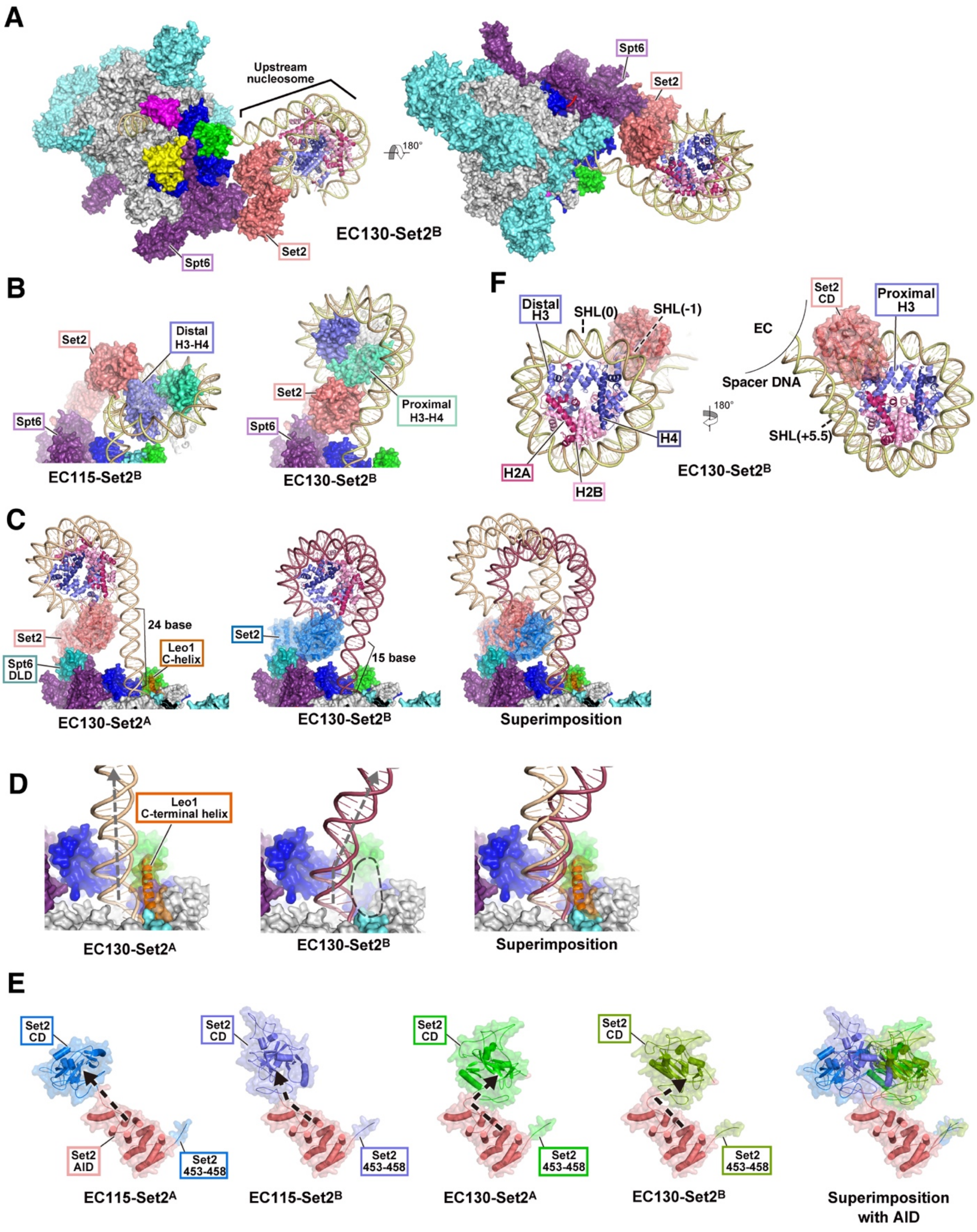

Fig. S17. Cryo-EM structure of EC130-Set2^{B}. (**A**) Overall structure of EC130-Set2^{B}. The EC and Set2 structures are shown in surface models. The nucleosome structure is shown in a ribbon model. (**B**) Comparison of the Set2-histone interactions between EC115 and EC130. The

promoter-distal H3-H4 and promoter-proximal H3-H4 in the DNA template are colored blue and cyan, respectively. Rtf1, a Paf1C subunit, is omitted for clarity. (**C**) Comparison of the upstream DNA in EC130-Set2^A and EC130-Set2^B. (**D**) Close-up views around the DNA exit tunnel. EC130-Set2^A (left), EC130-Set2^B (middle), and superimposition of these two structures (right) are shown. The Leo1 C-terminal helix is observed only in EC130-Set2^A. (**E**) Structural flexibility between Set2 CD and Set2 AID. Orientations of the hinge between Set2 CD and AID are shown as a dotted line. The superimposition is also shown. (**F**) The nucleosome-Set2 CD structures contained in EC-Set2^B. The CD is shown in a ribbon model with a transparent surface model.

Position	CDS Mutation	AA Mutation	Legacy Mutation ID	Count	Type
2024	c.6070C>T	p.R2024*	COSM1423518	2	Substitution - Nonsense
2024	c.6071G>A	p.R2024Q	COSM3823998	4	Substitution - Missense
2024	c.6071G>C	p.R2024P	COSM1045438	1	Substitution - Missense
2026	c.6076C>T	p.P2026S	COSM4502193	1	Substitution - Missense
2026	c.6077C>T	p.P2026L	COSM6981614	1	Substitution - Missense

Fig. S18. SETD2 mutations in cancer. Mutations of SETD2 2023-2027 residues in the COSMIC database (Aug. 5, 2024).

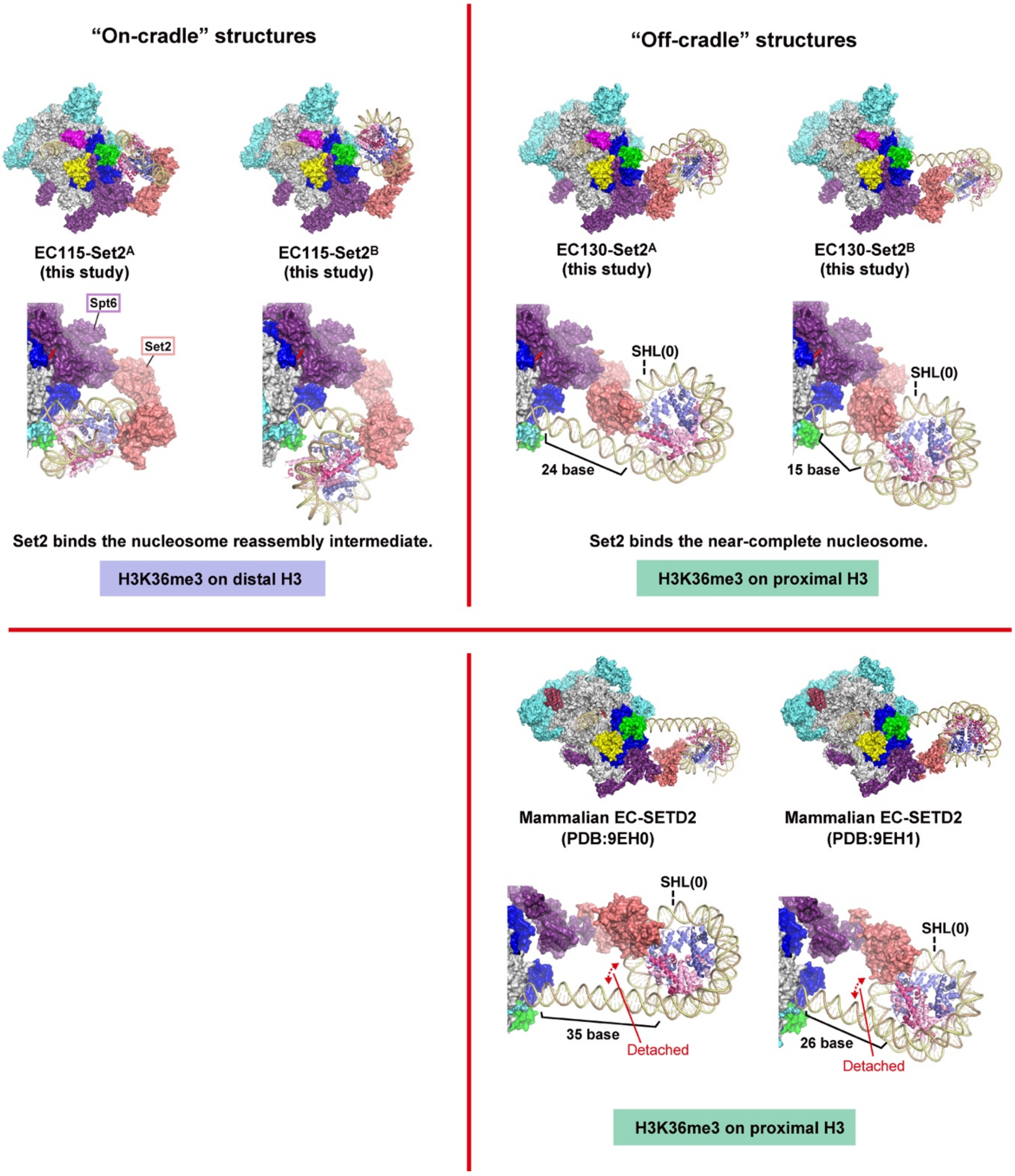

Fig. S19. Structural comparisons of EC-Set2/SETD2 structures. Overall structure of the EC-Set2/SETD2 structures and close-up views of the upstream regions of EC structures are shown. In the close-up views, Rtf1, a Paf1C subunit, is omitted for clarity.

Data collection statistics of EC115-Set2

**Sample**	**Temp115/ batch1**	**Temp115/ batch2**	**Temp115/ batch3**
Microscope	Krios G4 (RIKEN IMS)	Krios G4 (RIKEN IMS)	Krios G4 (RIKEN IMS)
Voltage (kV)	300	300	300
Detector	K3/BioQuantum	K3/BioQuantum(CDS)	K3/BioQuantum(CDS)
Slit width (eV)	15	15	15
Magnification	105,000	105,000	105,000
Pixel size for data collection (Å)	0.83	0.83	0.83
Total electron exposure (e^-/Å^2)	61.9	58.9	56.2
Exposure time (s)	2.2	4.3	4.3
Exposure rate (e^-/pixel/sec)	19.4	9.49	8.98
Number of frames	48	48	48
Defocus range (mm)	-1.2 to -2.0	-1.2 to -2.0	-1.2 to -2.0
Number of collected micrographs	13117	23113	26095

Table S1. Data collection statistics of EC115-Set2.

Refinement and model building statistics of EC115-Set2

Model	EC115-Set2^{A}	EC115-Set2^{B}	Spt6 in EC115-Set2	Spt6-Set2(AID) in EC115-Set2	Nucleosome-Set2(CD) in EC115-Set2	EC115^{hex}-Set2-FACT
EMDB ID	EMD-66104	EMD-66103	EMD-68371	EMD-68372	EMD-68373	EMD-66107
Number of particles	35823	59019	183297	23223	16189	29764
Pixel size for refinement (Å)	1.47	1.47	1.47	1.47	1.47	1.47
Symmetry imposed	C1	C1	C1	C1	C1	C1
Global resolutions (Å)						
Overall	3.59	3.37	3.06	3.77	4.11	3.62
Upstream	7.37	7.37	-	-	-	4.84
Nucleosome	4.11	3.96	-	-	-	3.81
PDB ID	9WMT	9WMS	-	-	-	9WMW
MolProbity score	1.12	1.13	-	-	-	1.18
Clash score	3.06	3.08	-	-	-	3.22
RMSDs			-	-	-	
Bond length (Å)	0.004	0.004	-	-	-	0.006
Bond angle (°)	0.706	0.696	-	-	-	0.830
Ramachandran plot (%)			-	-	-	
Outliers	0.01	0.01	-	-	-	0.03
Allowed	2.08	2.11	-	-	-	2.29
Favored	97.91	97.87	-	-	-	97.67
Rotamer outliers (%)	0.06	0.06	-	-	-	0.13

Table S2. Refinement and model building statistics of EC115-Set2.

Data collection statistics of EC115-Set2 without H2BK120Cub

**Sample**	**Temp115(noUb)/ batch1**	**Temp115(noUb)/ batch2**	**Temp115(noUb)/ batch3**
Microscope	Krios G4 (RIKEN IMS)	Krios G4 (RIKEN IMS)	Krios G4 (RIKEN IMS)
Voltage (kV)	300	300	300
Detector	K3/BioQuantum	K3/BioQuantum	K3/BioQuantum
Slit width (eV)	15	15	15
Magnification	105,000	105,000	105,000
Pixel size for data collection (Å)	0.83	0.83	0.83
Total electron exposure (e^-/Å^2)	59.5	59.4	59.0
Exposure time (s)	2.6	2.3	2.6
Exposure rate (e^-/pixel/sec)	15.9	15.8	15.7
Number of frames	48	48	48
Defocus range (mm)	-1.6 to -2.0	-1.6 to -2.0	-1.6 to -2.0
Number of collected micrographs	20322	11935	15265

Table S3. Data collection statistics of EC115-Set2 without H2BK120Cub.

Stain	Genotype	Source
BY4741	*MATa his3Δ1 leu2Δ0 met15Δ0 ura3Δ0*	ATCC: 201388
TKS4	*MATa his3Δ1 leu2Δ0 met15Δ0 ura3Δ0 set2Δ::kanMX6*	This study
TKS7	*MATa his3Δ1 leu2Δ0 met15Δ0 ura3Δ0 set2Δ::SET2-3xFLAG-URA3*	This study
TKS11	*MATa his3Δ1 leu2Δ0 met15Δ0 ura3Δ0 set2Δ::set2(5A)-3xFLAG-URA3*	This study
TKS20	*MATa his3Δ1 leu2Δ0 met15Δ0 ura3Δ0 set2Δ::set2(ΔSRI)-3xFLAG-URA3*	This study
TKS25	*MATa his3Δ1 leu2Δ0 met15Δ0 ura3Δ0 set2Δ::set2(5A-ΔSRI)-3xFLAG-URA3*	This study

Table S4. The *Saccharomyces cerevisiae* strains used in this study.

Data collection statistics of EC130-Set2

Sample	Temp130/ batch1	Temp130/ batch2	Temp130/ batch3	Temp130/ batch4
Microscope	Krios G4 (RIKEN IMS)	Krios G4 (RIKEN IMS)	Krios G4 (RIKEN IMS)	Krios G4 (RIKEN IMS)
Voltage (kV)	300	300	300	300
Detector	K3/BioQuantum	K3/BioQuantum	K3/BioQuantum	K3/BioQuantum
Slit width (eV)	15	15	15	15
Magnification	105,000	105,000	105,000	105,000
Pixel size for data collection (Å)	0.83	0.83	0.83	0.83
Total electron exposure (e^-/Å^2)	59.3	59.0	59.0	59.0
Exposure time (s)	2.6	2.6	2.6	2.6
Exposure rate (e^-/pixel/sec)	15.8	15.7	15.7	15.7
Number of frames	48	48	48	48
Defocus range (mm)	-1.2 to -2.0	-1.6 to -2.0	-1.6 to -2.0	-1.6 to -2.0
Number of collected micrographs	18905	17648	18500	14511

Table S5. Data collection statistics of EC130-Set2.

Refinement and model building statistics of EC130-Set2

**Model**	**EC130-Set2[A]**	**EC130-Set2[B]**
EMDB ID	EMD-66105	EMD-66106
Number of particles	15255	10768
Pixel size for refinement (Å)	1.47	1.47
Symmetry imposed	C1	C1
Global resolutions (Å)		
Overall	4.28	4.66
Upstream	3.82	4.12
Nucleosome	4.38	7.73
PDB ID	9WMU	9WMV
MolProbity score	1.11	1.10
Clash score	3.00	2.89
RMSDs		
Bond length (Å)	0.004	0.004
Bond angle (°)	0.708	0.707
Ramachandran plot (%)		
Outliers	0.01	0.01
Allowed	2.07	2.09
Favored	97.92	97.90
Rotamer outliers (%)	0.06	0.09

Table S6. Refinement and model building statistics of EC130-Set2.

Movie S1.

Structures of EC115-Set2^{A}, EC115^{hex}-Set2-FACT, and EC130-Set2^{A}.
